# Dereplication-Guided Isolation of New Phenylpropanoid-Substituted Diglycosides from *Cistanche salsa* and Their Inhibitory Activity on NO Production in Macrophage

**DOI:** 10.3390/molecules22071138

**Published:** 2017-07-08

**Authors:** Jongmin Ahn, Hee-Sung Chae, Young-Won Chin, Jinwoong Kim

**Affiliations:** 1 College of Pharmacy and Research Institute of Pharmaceutical Sciences, Seoul National University, Seoul 08826, Korea; jm212224@snu.ac.kr; 2 College of Pharmacy and Integrated Research Institute for Drug Development, Dongguk University-Seoul, Gyeonggi-do 10326, Korea; chaeheesung83@gmail.com (H.-S.C.); f2744@dongguk.edu (Y.-W.C.)

**Keywords:** dereplication, phenylpropanoid-substituted diglycosides, *Cistanche salsa*, anti-inflammatory

## Abstract

Dereplication allows for a rapid identification of known and unknown compounds in plant extracts. In this study, we performed liquid chromatography-mass spectroscopy (LC-MS)- based dereplication using data from ESI^+^ QTOF-MS for the analysis of phenylpropanoid-substituted diglycosides, the major active constituents of *Cistanche salsa* (C. A. Mey.) Beck. Using TOF-MS alone, the substructures of these compounds could be unambiguously confirmed based on the characteristic fragmentation patterns of various product ions. HPLC-MS based profiling of *C. salsa* also allowed for the detection of new phenylpropanoid-substituted diglycosides from this plant. Of them, five new phenylpropanoid-substituted diglycosides, named cistansalsides A–E (**5**, **6**, **12**, **17** and **18**), were isolated. Their structures were elucidated through spectroscopic methods including NMR and MS analysis. All the isolates were tested for their inhibitory activity against NO production in RAW 264.7 cells stimulated by LPS. Of the tested compounds, compounds **5**, **11**, **13** and **18** showed moderate inhibitory activity on inducible NO synthase. Compounds **11**, **13** and **18** also inhibited the phosphorylation of NF-κB in macrophages. None of the compounds displayed significant cytotoxicity.

## 1. Introduction

*Cistanche salsa* (C. A. Mey.) Beck, belonging to the family Orobanchaceae, is a parasitic plant that obtains its nutrition from the root of *Haloxylon ammodendron* (Chenopodiaceae) and other desert plants [1]. This plant has been used in traditional medicine for the treatment of neurasthenia, sexual dysfunction and kidney deficiency [2,3]. In previous phytochemical studies, it has been reported that the whole plant of *C. salsa* contained various types of compounds including phenylethanoid glycosides and iridoid glycosides [4,5,6,7]. Phenylethanoid glycosides, such as acteoside and echinacoside, are the major active constituents of the plant [8]. The extracts of *C. salsa* showed beneficial properties, including immunomodulatory, anticancer and antiinflammatory activities [9,10].

Dereplication is a process by which sample mixtures would be tested to differentiate unknown constituents from known compounds. The dereplication strategies are based on the analytical techniques and database searching to identify secondary metabolites early in the phytochemical research process [11]. Of the analytical techniques, ESI-QTOF-MS (electrospray ionization-quadrupole-time of flight-mass spectroscopy) could provide valuable information about chemical structures of secondary metabolites. The LC-MS-based dereplication-guided fractionation has been demonstrated to enable extraction and purification of target metabolites from crude extracts of plants with high efficiency [12,13,14,15].

**Figure 1:**
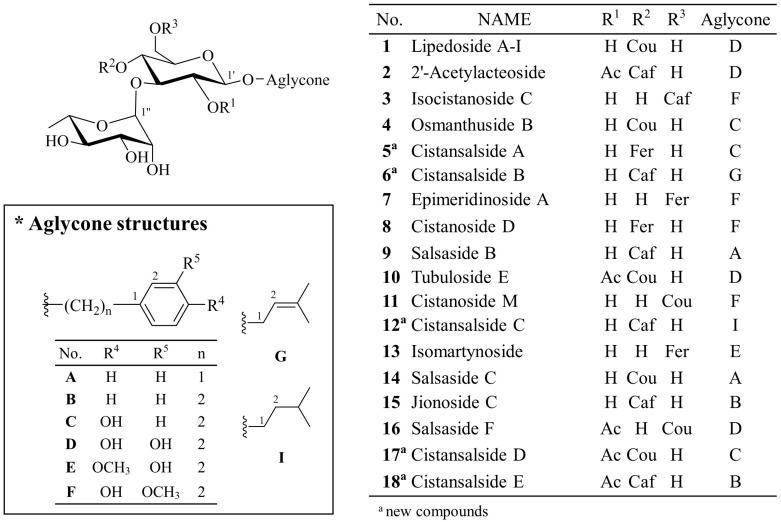
Structures of compounds **1**–**18**.

This study performed the LC-MS-based dereplication using data from ESI^+^ TOF-MS for analysis of phenylpropanoid-substituted diglycosides, the major active constituents of *C. salsa*. The TOF-MS data could suggest the substructures of these compounds based on characteristic fragmentation patterns of various product ions. Based on this dereplication, LC-MS profiles of ethyl acetate (EtOAc) fraction and water-soluble fraction were analyzed. The EtOAc fraction was subjected to the dereplication strategy for further separation, resulted in the isolation of five new phenylpropanoid-substituted diglycosides and 13 known compounds (Figure 1). It was confirmed that tentatively predicted structures of phenylpropanoid-substituted diglycosides were correctly matched to their real structures. In addition, the anti-inflammatory activities of the isolates were explored.

## 2. Results and Discussion

The phenylpropanoid-substituted diglycosides isolated from *C. salsa* usually have structures based on disaccharide glycosides, which consist of a glucose and a rhamnose with a Rha (1→3) Glc linkage and one cinnamoyl substituent, such as coumaric acid (Cou), caffeic acid (Caf) and feruloyl acid (Fer), at the C-4 or C-6 position of glucose. The aglycone is commonly attached at the C-1 position of glucose. The structures of phenylpropanoid-substituted diglycosides with an acetyl group at the C-2 of glucose have frequently been reported [6,12,16].

**Figure 2:**
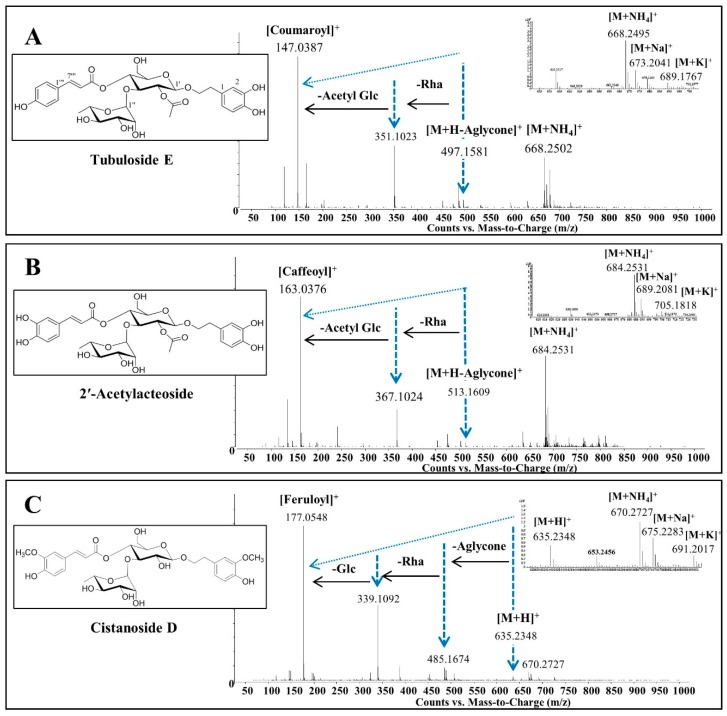
The fragmentation pathways of phenylpropanoid-substituted diglycosides. (**A**) Tubuloside E, C_31_H_38_O_15_, M.W. 650; (**B**) 2′-Acetylacteoside, C_31_H_38_O_16_, M.W. 666; (**C**) Cistanoside D, C_31_H_40_O_15_, M.W. 652

To perform the dereplication, MS fragmentation patterns of these compounds were analyzed by positive mode ESI-QTOF-MS. In MS spectra, all the phenylpropanoid-substituted diglycosides produced adduct ion peaks at [M + NH_4_]^+^, [M + K]^+^ and [M + Na]^+^, which provided the molecular weight and formula. The pattern of fragment ions could be found by successive losses of aglycone and glycoside residues ([M + H − Aglycone]^+^, [M + H − Aglycone − Rha]^+^ and [M + H − Aglycone – Rha − Glc (or Acetyl-Glc)]^+^), which were useful for predicting the type of cinnamoyl substituent and sugars. The fragment ions at *m/z* 163 of the caffeoyl group, *m/z* 147 of the coumaroyl group or *m/z* 177 of the feruloyl group give the characteristic signal of a cinnamoyl substituent in the phenylpropanoid-substituted diglycosides [4,15] (Figure 2). The analysis of the fragment ions would provide useful information for the identification of the structures of phenylpropanoid-substituted diglycosides. However, their isomers could not be differentiated by MS spectrometry alone. For accurate identification of their complete structures, NMR spectra are required.

**Figure 3:**
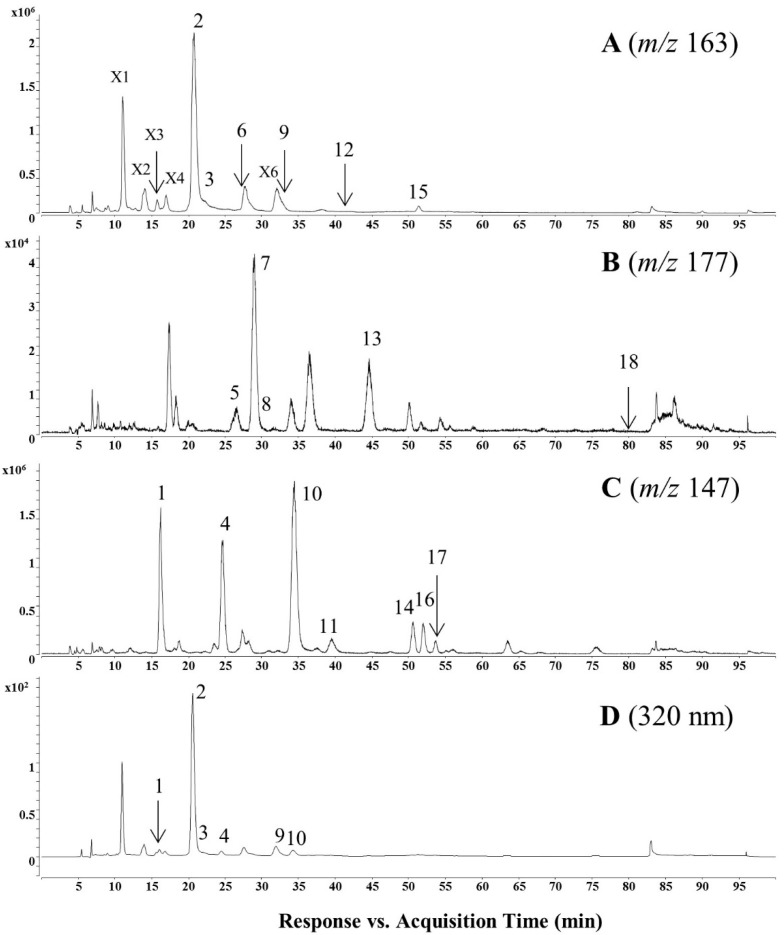
Base peak chromatogram and extracted ion chromatograms of EtOAc fraction of *C. salsa* analyzed by HPLC-ESI-QTOF-MS in positive mode: (**A**) Caf at *m/z* 163; (**B**) Fer at *m/z* 177; (**C**) Cou at *m/z* 147; (**D**) base peak chromatogram at 320 nm.

*C. salsa* was analyzed and the fingerprint of the EtOAc fraction was generated using the HPLC-DAD (diode array detector)-ESI-QTOF-MS method (Figure 3). Each peak in the fingerprint of *C. salsa* was predicted according to MS fragmentation features (Table 1). Many phenylpropanoid-substituted diglycosides were screened out from this fraction, which was subjected to HPLC-QTOF-MS-guided isolation for the discovery of new phenylpropanoid-substituted diglycosides. Eighteen peaks including five new compounds were further identified and their structures were elucidated through extensive spectroscopic analysis. 

Cistansalside A (**5**) was obtained as a brown amorphous powder. Its molecular formula was determined to be C_30_H_38_O_14_ by positive mode high resolution (HR) ESI-QTOF-MS based on the adduct ion peak at *m/z* 645.2146 [M + Na]^+^ (calcd. for C_30_H_38_O_14_Na, 645.2154). A characteristic ion at *m/z* 177 suggested that a feruloyl substituent existed in its structure. The fragment ions at *m/z* 339 and *m/z* 485 suggested the presence of a rhamnose unit and a glucose unit as well.

The ^13^C-NMR spectrum showed 30 carbon atoms. Analysis of the ^1^H and HSQC spectra indicated the presence of two anomeric protons at δ_H_ 4.35 (1H, d, *J =* 7.9 Hz, H-1′) and 5.02 (1H, s, H-1′′) and a methoxy proton at δ_H_ 3.80 (3H, s, 3′′′-OCH_3_). The ^1^H-NMR spectrum showed an 1,3,4-trisubstituted benzene ring at δ_H_ 7.29 (1H, d, *J =* 1.3 Hz, H-2′′′), 7.09 (1H, dd, *J =* 8.3, 1.3 Hz, H-6′′′) and 6.79 (1H, d, *J =* 8.3 Hz, H-5′′′), a *trans*-olefin group at δ_H_ 7.53 (1H, d, *J =* 15.9 Hz, H-7′′′) and 6.41 (1H, d, *J =* 15.9 Hz, H-8′′′) and a *para*-substituted benzene ring at δ_H_ 7.05 (2H, d, *J =* 8.3 Hz, H-2, 6) and 6.67 (2H, d, *J =* 8.3 Hz, H-3, 5) (Table 2). 

A 3,4-dihydroxyphenyl group was suggested by the HMBC correlations between H-2′′′ and a quaternary aromatic carbon at δ_C_ 149.4 (C-4′′′) and between H-5′′′ and C-1′′′ (δ_C_ 125.6) and C-3′′′ (δ_C_ 147.9). From the HMBC NMR spectrum, the correlations between a carbonyl carbon at δ_C_ 165.8 and H-8′′′ and between H-6′′′ and C-7′′′ (δ_C_ 145.5) suggested a 3,4-dihydroxylated cinnamoyl group. The HMBC correlation between the methoxy proton and C-3′′′ and the NOESY correlation between the methoxy proton and H-2′′′ confirmed the cinnamoyl substituent to be an (*E*)-feruloyl group.

The 4-hydroxyphenyl group was suggested by the HMBC correlations between H-3,5 and quaternary aromatic carbons at δ_C_ 155.7 (C-4) and 128.5 (C-1). A hydroxyethyl group was confirmed by the COSY correlations among H-7 (2H, δ_H_ 2.76, m), H-8a (1H, δ_H_ 3.90, m) and H-8b (1H, δ_H_ 3.61, m). From the HMBC NMR spectrum, the correlation between C-7 (δ_C_ 34.7) and H-2, 6 suggested a 4-hydroxyphenylethyl group, as an aglycone substituent. 

**Figure 4:**
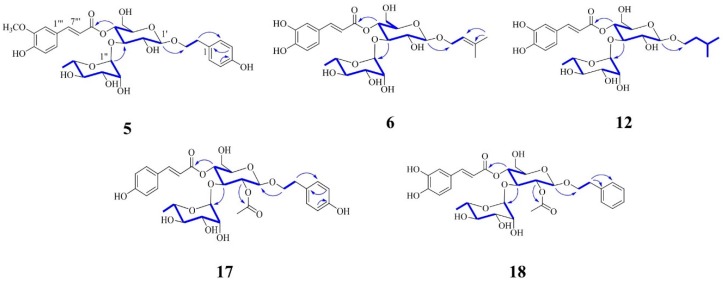
Key ^1^H-^1^H COSY (**bold line**) and HMBC (**blue arrow**) correlations of new compounds.

Two sugar moieties, suggested by the MS fragment pattern, were double-checked by the NMR spectra and HPLC analysis of the acid hydrolysate. The absolute configurations of them were determined to be d-glucose and l-rhamnose using HPLC analysis of the acid hydrolysate [17]. A β-glucose moiety and an *α*-rhamnose moiety were established by coupling constants of the anomeric protons. The ^1^H-^1^H COSY spectrum showed the sequential correlations from H-1′ to H-5′ and from H-1′′ to H-6′′ (Figure 4). 

From the ^1^H-NMR spectrum, the downfield shift of H-4′ (δ_H_ 4.71) suggested an acyl-substituent on glucose. The HMBC correlation between H-4′ and C-9′′′ (δ_C_ 165.8) confirmed that a feruloyl substituent was located at the C-4′ position. The aglycone was located at C-1′ according to the HMBC correlation between H-1′ and C-8 (δ_c_ 70.2). The HMBC correlation between H-1′′ and C-3′ (δ_C_ 79.2) gave us the position of rhamnose in this structure. Thus, the structure of **5** was established to be 4-hydroxyphenylethyl-*O*-*α*-l-rhamnopyranosyl-(1→3)-4-*O*-(*E*)-feruloyl-*β*-d-glucopyranoside and the compound was named cistansalside A.

Cistansalside B (**6**) was obtained as a brown amorphous powder. Its molecular formula was determined to be C_26_H_36_O_13_ based on the ^13^C-NMR data and a (+)-HR-ESI-QTOF-MS peak at *m/z* 579.2054 [M + Na]^+^ (calcd. for C_26_H_36_O_13_Na, 579.2048). Fragment ions including a [M + H − Aglycone − Rha − Glc]^+^ ion at *m/z* 163, a [M + H − Aglycone − Rha]^+^ ion at *m/z* 325 and a [M + H − Aglycone]^+^ ion at *m/z* 471 were also detected. A characteristic ion at *m/z* 163 suggested that a caffeoyl group existed in the structure.

The ^1^H-NMR spectrum showed an 1,3,4-trisubstituted benzene ring at δ_H_ 7.02 (1H, s, H-2′′′), 6.97 (1H, d, *J =* 8.3 Hz, H-6′′′) and 6.75 (1H, d, *J =* 8.3 Hz, H-5′′′), a *trans*-olefin at δ_H_ 7.47 (1H, d, *J =* 15.8 Hz, H-7′′′) and 6.19 (1H, d, *J =* 15.8 Hz, H-8′′′). The ^1^H and HSQC NMR spectra showed two anomeric protons at δ_H_ 4.29 (1H, d, *J =* 7.9 Hz, H-1′) and 5.01 (1H, s, H-1′′), an olefinic proton at δ_H_ 5.13 (1H, dd, *J =* 7.6, 6.5 Hz, H-2), two methylene protons at δ_H_ 4.23 (1H, dd, *J =* 12.2, 6.5 Hz, H-1a) and 4.13 (1H, dd, *J =* 12.2, 7.6 Hz, H-1b) and germinal methyl groups at δ_H_ 1.71 (3H, s, H-4) and 1.63 (3H, s, H-5) (Table 2).

From the HMBC spectrum, the 3,4-dihydroxyphenyl group was suggested by the correlations between H-2′′′ and a quaternary aromatic carbon at δ_C_ 149.0 (C-4′′′) and between H-5′′′ and another quaternary carbons at δ_C_ 125.5 (C-1′′′) and 148.5 (C-3′′′). From the HMBC NMR spectrum, the correlations between H-6′′′ and C-7′′′ (δ_C_ 145.6) and between the carbonyl carbon at δ_c_ 165.7 (C-9′′′) and H-8′′′ suggested an (*E*)-caffeoyl group. 

A 3-methylbutenyl group, an aglycone substructure, was suggested by the COSY correlations of H-2 with H-1a and H-1b and the HMBC correlations between H-4 and H-5 and the olefinic carbons, at δ_C_ 120.7 (C-2) and 136.4 (C-3). 

Two sugar moieties were established by the NMR spectra analysis and HPLC spectra analysis of the acid hydrolysate, with MS fragment pattern. The absolute configuration of them were determined to be d-glucose and l-rhamnose using HPLC analysis of the acid hydrolysate [17]. These sugar moieties were defined as a β-glucose and an *α*-rhamnose by coupling constants of the anomeric protons. The ^1^H-^1^H COSY spectrum showed the sequential correlations from H-1′ to H-6′ and from H-1′′ to H-6′′ (Figure 4). 

A downshifted glucose proton at δ_H_ 4.70 (H-4′) suggested an acyl-substituent on glucose. From the HMBC spectrum, the correlation between H-4′ and C-9′′′ confirmed the position of caffeoyl substituent. The HMBC correlations between H-1′ and C-1 (δ_C_ 64.5) and between H-3′ (δ_H_ 3.68) and C-1′′ (δ_C_ 101.2) suggested the positions of each substituents. Accordingly, the structure of **6** was determined as 3-methylbutenyl-*O*-α-l-rhamnopyranosyl-(1→3)-4-*O*-(*E*)-caffeoyl-β-d-gluco-pyranoside and named cistansalside B.

Cistansalside C (**12**), a brown amorphous powder, was determined to have a molecular formula of C_26_H_38_O_13_ by (+)-HR-ESI-QTOF-MS, which showed a peak at *m/z* 581.2213 [M + Na]^+^ (calcd. for C_26_H_38_O_13_Na, 581.2205). A characteristic ion at *m/z* 163 suggested that a caffeoyl substituent existed in the structure. The fragment ions at *m/z* 325 and *m/z* 471 suggested the existence of a rhamnose unit and a glucose unit.

Comparison of the NMR spectra of **12** with those of **6** showed that they were similar except for the aglycone structure. In the NMR spectra of **12**, two paraffinic carbons at δ_C_ 38.0 (C-2) and 24.4 (C-3) were observed instead of two olefinic carbons at δ_C_ 120.7 (C-2) and 136.4 (C-3) in the aglycone of **6**. The germinal methyl groups (δ_H_ 0.88) in the aglycone of **12** were shifted upfield relative to H-4 and H-5 (δ_H_ 1.71 and 1.63) in the aglycone of **6** (Table 2). The aglycone of **12** was suggested to be a 3-methylbutyl group, which was confirmed by the ^1^H and COSY NMR spectra. Peaks of 3-methylbutyl group were observed at δ_H_ 3.81 (1H, m, H-1a), 3.48 (1H, m, H-1b), 1.71 (1H, m, H-3), 1.44 (2H, m, H-2) and 0.88 (H-4, 5). 

Two sugar moieties were reaffirmed by the HPLC analysis of the acid hydrolysate and the NMR spectra analysis as well as MS fragment pattern. The absolute configurations of the sugars were identified as d-glucose and l-rhamnose using HPLC analysis of the acid hydrolysate [17]. A β-glucose moiety and an *α*-rhamnose moiety were confirmed by coupling constants of the anomeric protons. The ^1^H-^1^H COSY spectrum showed the sequential correlations from H-1′ to H-5′, from H-1′′ to H-3′′ and from H-6′′ to H-4′′ (Figure 4). 

The position of substituents were confirmed by means of the HMBC analysis. In the HMBC spectrum, the correlations between H-1′ and C-1 (δ_C_ 67.2), between H-3′ (δ_H_ 3.68) and C-1′′ (δ_C_ 101.2) and between H-4′ (δ_H_ 4.70) and C-9′′′ (δ_C_ 165.7) were detected. Consequently, the structure of **12** was established to be 3-methylbutyl-*O*-*α*-l-rhamnopyranosyl-(1→3)-4-*O*-(*E*)-caffeoyl-β-d-gluco-pyranoside and named cistansalside C.

Cistansalside D (**17**), an amorphous brown powder, was determined to have a molecular formula of C_31_H_38_O_14_ by the positive mode high-resolution ESI-QTOF-MS, which showed an adduct ion peak at *m/z* 657.2147 [M + Na]^+^ (calcd. for C_31_H_38_O_14_Na, 657.2154). Fragment ions including a [M + H − Aglycone − Rha − Acetyl Glc]^+^ ion at *m/z* 147, a [M + H − Aglycone − Rha]^+^ ion at *m/z* 351 and a [M + H − Aglycone]^+^ ion at *m/z* 497 were also detected. A characteristic ion at *m/z* 147 suggested that a coumaroyl substituent was present in its structure. The fragment ions at *m/z* 351 and *m/z* 497 suggested the existence of a rhamnose unit and an acetyl-substituted glucose unit. 

The ^13^C-NMR spectrum revealed the presence of 31 carbon atoms. Two anomeric protons at δ_H_ 4.61 (1H, m, H-1′) and 4.60 (1H, s, H-1′′) were observed in the ^1^H and HSQC spectra. The ^1^H-NMR spectrum indicated the presence of a methyl group at δ_H_ 1.97 (3H, s, acetyl-CH_3_), a *trans*-olefin at δ_H_ 7.55 (1H, d, *J =* 15.9 Hz, H-7′′′) and 6.34 (1H, d, *J =* 15.9 Hz, H-8′′′) and two *para*-substituted benzene rings at δ_H_ 7.53 (2H, d, *J =* 6.9 Hz, H-3′′′, 5′′′) and 6.79 (2H, d, *J =* 6.9 Hz, H-2′′′, 6′′′)/ δ_H_ 6.98 (2H, d, *J =* 8.0 Hz, H-2, 6) and 6.65 (2H, d, *J =* 8.0 Hz, H-3, 5) (Table 2). From the HMBC NMR spectrum, the correlations between a carbonyl carbon at δ_c_ 165.5 (C-9′′′) and H-8′′′ and between H-2′′′, 6′′′ and C-7′′′ (δ_c_ 145.4) suggested an (*E*)-coumaroyl group. The HMBC correlation between a methyl proton peak and a carbonyl carbon at δ_c_ 169.0 confirmed the presence of an acetyl group. 

A 4-hydroxyphenyl group was suggested based on the HMBC correlations between H-3, 5 and quaternary aromatic carbons at δ_C_ 155.6 (C-4) and 128.6 (C-1). A hydroxylated ethyl group was confirmed by the COSY NMR signals at δ_H_ 2.66 (2H, t, *J =* 6.1 Hz, H-7), 3.90 (1H, m, H-8a) and 3.54 (1H, m, H-8b). The HMBC correlations between H-7 and C-1 (δ_C_ 128.6) and C-2, 6 (δ_C_ 129.7) suggested a 4-hydroxyphenylethyl group, as an aglycone substructure. 

Two sugar moieties, suggested from MS fragment pattern, were reconfirmed by the HPLC analysis of the acid hydrolysate and NMR spectra. d-glucose and l-rhamnose were elucidated using HPLC analysis of the acid hydrolysate [17]. A β-glucose moiety and an *α*-rhamnose moiety were established by coupling constants of the anomeric protons. The ^1^H-^1^H COSY spectrum showed the sequential correlations from H-1′ to H-5′ and from H-1′′ to H-6′′ (Figure 4). 

From the ^1^H-NMR spectrum, the downfield shift of H-2′ (δ_H_ 4.69) and H-4′ (δ_H_ 4.80) suggested the acyl-substituted position on glucose. The connections between glucose and two acyl groups were confirmed by the HMBC correlations between H-4′ (δ_H_ 4.80) and C-9′′′ and between H-2′ and a carbonyl carbon of an acetyl group (δ_C_ 169.0). The positions of an aglycone and a rhamnose were given by the HMBC correlations between H-3′ (δ_H_ 3.95) and C-1′′′ and between H-8 and C-1′. Accordingly, the structure of **17** was assigned as 4-hydroxyphenylethyl-2-*O*-acetyl-*O*-α-l-rhamnopyranosyl-(1→3)-4-*O*-(*E*)-coumaroyl-β-d-glucopyranoside and named cistansalside D.

Cistansalside E (**18**) was isolated as an amorphous brown powder. Its molecular formula was determined to be C_31_H_38_O_14_ by ^13^C-NMR data and the positive mode high-resolution ESI-QTOF-MS peak at *m/z* 657.2166 [M + Na]^+^ (calcd. for C_31_H_38_O_14_Na, 657.2154). Additionally, fragment ions including a caffeoyl ion at *m/z* 163, an [M + H − Aglycone − Rha]^+^ ion at *m/z* 367 and an [M + H − Aglycone]^+^ ion at *m/z* 513 were also detected. The fragment ions suggested the existence of a rhamnose unit and an acetyl-substituted glucose unit.

The ^1^H-NMR spectrum suggested the presence of two anomeric protons at δ_H_ 4.63 (1H, d, *J =* 8.2 Hz, H-1′) and 4.60 (1H, s, H-1′′) and two acyl-substituted glucose protons at δ_H_ 4.71 (1H, dd, *J =* 8.9, 8.2 Hz, H-2′) and 4.81 (1H, dd, *J =* 9.7, 9.5 Hz, H-4′). The ^1^H and HMBC spectra suggested the existence of an acetyl group at δ_H_ 1.94 (3H, s, acetyl-CH_3_), an (*E*)-caffeoyl moiety at δ_H_ 7.48 (1H, d, *J =* 15.9 Hz, H-7′′′), 7.02 (1H, d, *J =* 1.6 Hz, H-2′′′), 6.98 (1H, dd, *J =* 8.1, 1.6 Hz, H-6′′′), 6.76 (1H, d, *J =* 8.1 Hz, H-5′′′) and 6.21 (1H, d, *J =* 15.9 Hz, H-8′′′) and a mono-substituted benzene ring at δ_H_ 7.29 (2H, m, H-3, 5), 7.21 (2H, m, H-2, 6) and 7.20 (1H, m, H-4) (Table 2). A phenylethyl group, an aglycone substructure, was suggested by the HMBC correlations between H-7 (2H, δ_H_ 2.80, m) and C-1 (δ_C_ 138.8) and between H-7 and C-2, 6 (δ_C_ 128.9) and the COSY NMR signals of H-7 with H-8a (1H, δ_H_ 3.99, m) and H-8b (1H, δ_H_ 3.63, m). 

Two sugar moieties were reaffirmed by the HPLC analysis of the acid hydrolysate and NMR spectra analysis. The absolute configurations of the sugars were elucidated using HPLC analysis of the acid hydrolysate, which were confirmed to be d-glucose and l-rhamnose [17]. A β-glucose moiety and an α-rhamnose moiety were established by coupling constants of the anomeric protons. The ^1^H-^1^H COSY spectrum showed the sequential correlations from H-1′ to H-5′, from H-1′′ to H-2′′ and from H-6′′ to H-3′′ (Figure 4). 

From the HMBC spectrum, the correlations between H-1′ and C-8 (δ_C_ 69.4), between H-2′ and a carbonyl carbon of an acetyl group (δ_C_ 169.1), between H-3′ (δ_H_ 3.95) and C-1′′ (δ_C_ 102.0) and between H-4′ (δ_H_ 4.81) and C-9′′′ (δ_C_ 165.6) confirmed the position of substituents in the structure. Therefore, the structure of **18** was determined to be phenylethyl-2-*O*-acetyl-*O*-*α*-l-rhamnopyranosyl-(1→3)-4-*O*-(*E*)-caffeoyl-β-d-glucopyranoside and named cistansalside E.

Most of the *trans*-cinnamoyl substituents were isomerized to the *cis*-isoform in vitro. Light has been reported to convert *trans*-cinnamic acid derivatives into *cis*-isoforms [18,19]. The equilibrium of the *trans*-*cis* conversion of the cinnamoyl substituents was observed to maintain approximately 70% of the isolates in the *trans*-isoform. For the olefin protons of the *cis* form, peaks at approximately 6.90 ppm (d, *J* = 12~13 Hz, H-7′′′) and 5.80 ppm (d, *J* = 12~13 Hz, H-8′′′) were assignable in the ^1^H-NMR spectra, whereas peaks at approximately 7.55 ppm (d, *J* = 15.8 Hz, H-7′″) and 6.40 ppm (d, *J* = 15.8 Hz, H-8′′′) were observed for *trans* form [20]. In the ^1^H-NMR spectrum of *trans*-*cis* mixtures, peaks for two olefinic protons (H-7′′′ and H-8′′′) were observed in the ratio of 7:3 (*trans*:*cis*). ^13^C-NMR peaks of the *cis* form were similar to those of the *trans* form. 

**Figure 5:**
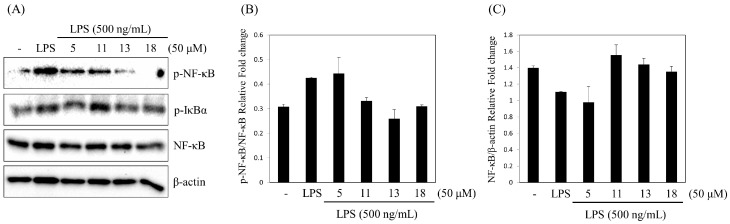
Effect of four compounds on phosphorylation of IκBα and NF-κB (p65) in LPS-stimulated RAW 264.7 cells. (**A**) The western blots were conducted in LPS and sample-treated RAW 264.7 cells; (**B**,**C**) The immunoblot signals were quantified using Molecular Analyst/PC densitometry software (Bio-Rad, Richmond, CA, USA). Densitometric analysis of phosphorylated isoforms is reported. NF-κB in RAW 264.7 cell was normalized to the content of β-actin.

All the isolates were tested for their inhibitory effects on LPS-induced NO production in RAW 264.7 cells. Dexamethasone was used as a positive control and its IC_50_ was 7.0 μM. Of the tested compounds, compounds **5** (IC_50_ 42.7 ± 6.6 μM), **11** (IC_50_ 37.3 ± 2.2 μM), **13** (IC_50_ 40.0 ± 4.0 μM) and **18** (IC_50_ 27.9 ± 0.8 μM) showed moderate inhibitory activities on inducible NO synthase, while the other compounds were inactive in this assay (IC_50_ values > 100 μM). To verify whether these compounds had cytotoxicity, cell viability was measured employing MTT assay. As a result, none of them displayed significant cytotoxicity (Supplemental Figure S6-1). These four compounds were selected to evaluate for their inhibitory activity against NF-κB pathway in LPS-stimulated RAW 264.7 cells. Stimulation of RAW 264.7 cells with LPS induced the phosphorylation of IκBα and NF-κB (p65) after 0.5 h of incubation. The phosphorylation of NF-κB (p65) was significantly reduced by pretreatment with compounds **11**, **13** and **18** as shown by western blot analysis (Figure 5). Therefore, compounds **11**, **13** and **18** might exert anti-inflammatory effects via the inhibition of NF-κB in macrophages.

## 3. Materials and Methods 

### 3.1. General Experiment Procedure

Optical rotations were measured with a Jasco P-2000 digital polarimeter (Jasco, Tokyo, Japan). UV spectra were recorded on a Chirascan plus Circular Dichroism spectrometer (Chirascan, APL, UK). IR spectra were recorded using Jasco FT/IR-4200 spectrophotometer. High-resolution electrospray ionization quadrupole-time-of-flight mass spectrometry (HR-ESI-qTOF-MS) was performed on an Agilent 6530 Accurate-Mass Q-TOF LC/MS equipped with Agilent 1260 Infinity series (Agilent Technologies, Inc., Palo Alto, CA, USA), and the column used was a Jasco SFCpak Crest C18T-5 column (i.d. 150 × 4.6 mm, 5 μm) . MassHunter Workstation Software was used for data acquisition.

1D (^1^H and ^13^C) and 2D (^1^H-^1^H COSY, HSQC, HMBC, NOESY) NMR spectra were obtained with a Jeol LA 300 (Jeol, Tokyo, Japan), Bruker AVANCE-400, Bruker AVANCE-500, Bruker AVANCE-600 and Bruker AVANCE 800 HD spectrometers coupled with cryoprobe (Bruker, Ettlingen, Germany). DMSO-*d_6_* (Cambridge Isotope Laboratories, Inc. Andover, MA, USA) was used as NMR solvent and reference peaks (δ_H_ 2.50 and δ_C_ 39.5). Column chromatography (CC) was performed using Sephadex LH-20 (25–100 μm; Pharmacia, Uppsala, Sweden) or Kieselgel 60 silica gel (40–63 μm, 230–400 mesh, Art. 9385; Merck, Darmstadt, Germany). Thin-layer chromatography (TLC) was conducted on pre-coated Kieselgel 60 silica gel F_254_ plates (Art. 5715; Merck). Spots on TLC were detected using UV lamp at 254 nm and 365 nm (VL-4.LC, 365/254; Vilber Lourmat, Torcy, France). Medium pressure liquid chromatography (MPLC) was performed on a RediSep 120 g silica flash column (Isco, Lincoln, NE, USA) and Kiesegel 60 silica gel (40–63 μm, 230–400 mesh, Art. 9385; Merck) using a Combiflash companion (Isco). The high pressure liquid chromatography (HPLC) system was a Gilson HPLC equipped with a Gilson 321 pump and UV.VIS 151 detector (Gilson, Middleton, WI, USA), using semi-preparative ODS columns (Luna 5 μm C18 (2) 100 Å, i.d. 250 × 10 mm, 5 μm, Phenomenex Inc., Torrance, CA, USA; Hypersil GOLD™ aQ 175 Å, i.d. 250 × 10 mm, 5 μm, Thermo Scientific™, Hennigsdorf, Germany; Inno C18 column 120 Å, i.d. 250 × 10 mm, 5 μm, Young Jin Biochrom Co., Ltd., Seongnam, Korea). The analytical RP-HPLC system was a Waters 2695 alliance system with a 996 Photodiode Array (PDA) detector (Waters Corp, Milford, MA, USA), and the column used was a Hypersil™ BDS C18 column (130 Å, i.d. 150 × 4.6 mm, 5 μm, Thermo Scientific™). Formic acid was purchased from Daejung Chemicals & Metals Co., Ltd. (Seoul, Korea). HPLC grade solvents were purchased from Fisher Scientific Korea Ltd. (Seoul, Korea). H_2_SO_4_, Na_2_CO_3_ and first grade solvents for extraction, fractionation and isolation were purchased from Daejung Chemical & Metals Co. Ltd. (Seoul, Korea). l- and d-cysteine methyl ester hydrochloride and *o*-tolylisothiocyanate were purchased from Tokyo Chemical Industry (Tokyo, Japan).

### 3.2. Plant Material

The whole plants of *C. salsa*, which were collected from the Shinjang Uyghur, were imported through Daerim Pharmaceutical Wholesale Company (Cheongju, Korea). They were identified by Prof. Dr. Jehyun Lee (Dongguk University, Seoul, Korea). The voucher specimen (SNUPH2016-03) was deposited in the Herbarium of Medicinal Plant Garden, College of Pharmacy, Seoul National University.

### 3.3. Extraction and Isolation

The dried whole plants of *C. salsa* (5.7 kg) were chopped and extracted three times with MeOH (20 L) at room temperature with sonication for 99 min. After removal of the solvent *in vacuo*, The crude extract (1.35 kg) was suspended in H_2_O (5 L), then partitioned with EtOAc (5 L). The EtOAc residue (55.3 g) was separated into 16 fractions (E01-16) on silica gel chromatography eluting with CHCl_3_/MeOH (50:1–0:1, step-gradient system). 

E08 (611.7 mg) was subjected to silica gel medium-pressure liquid chromatography (25 g) and eluted with CHCl_3_/MeOH (18:1–0:1, step-gradient system) and gave 11 fractions (E08a-k). From E08g, compounds **13** (0.7 mg) and **18** (0.6 mg) were purified using a Luna 5 μm HPLC column and isocratic elution with 28% aq. MeCN. HPLC purification (Hypersil GOLD, 25% aq. MeCN) of E08h (138.5 mg) furnished compounds **7** (10.6 mg), **8** (17.2 mg), **14** (13.4 mg) and **17** (4.9 mg). 

E09 (1.15 g) was further purified on a silica-MPLC column (20 g) eluting with CHCl_3_/MeOH (10:1–0:1, step-gradient system) to give 11 subfractions (E09a-k). E09e (398.7 mg) was subjected to Sephadex LH-20 (MeOH) and yielded eight subfractions (E09e1-8). E09e6 was subsequently purified using a Hypersil GOLD HPLC column (22% aq. MeCN) to afford compound **11** (0.4 mg). From E09e7, compound **5** (0.6 mg) was purified by isocratic eluction from a Luna 5 μm HPLC column with 40% aq. MeOH. 

E10 (1.20 g) was separated into nine fractions (E10a-i) on Sephadex LH-20 column eluting with MeOH. From E10g (147.5 mg), compounds **4** (13.4 mg), **9** (8.0 mg) and **15** (5.0 mg) were isolated by HPLC separation (Inno, 23% aq. MeCN). Fraction E10h (470.0 mg) was purified on a Hypersil GOLD column by isocratic elution (40% aq. MeOH) to yield compounds **1** (3.8 mg), **10** (42.1 mg) and **16** (3.0 mg). 

E11 (2.96 g) was subjected to Sephadex LH-20 eluting with MeOH to give nine fractions (E11a-i). E11e was separated by Sep-Pak C18 cartridge eluting stepwise with 10%, 20%, 30%, 50% and 100% aq. MeOH to yield seven fractions (E11e1-7), followed by Luna 5 μm HPLC (28% aq. MeCN) to give compounds **3** (3.4 mg), **6** (1.4 mg) and **12** (1.2 mg). 

E12 (19.0 g) was subjected to silica MPLC (120 g) using a CHCl_3_/MeOH step-gradient system to give six fractions (18:1–0:1, E12a-f). E12f was chromatographed on a Sephadex LH-20 column (MeOH), yielding seven fractions (E12f1-7). HPLC purification (Hypersil GOLD, 25% aq. MeCN) of E12f7 furnished compound **2** (3.3 mg). All solvents used for HPLC were 0.05% formic acid buffer. The common flow rate for HPLC and MPLC chromatography was 3 and 40 mL/min, respectively.

### 3.4 Characterization

*Cistansalside A* (**5**): brown amorphous powder; ${\left[\alpha \right]}_{D}^{20}$ −33.7 (*c* 0.1, MeOH); UV(MeOH) λ_max_ nm (log ε) 332 (3.18); IR (neat) ν_max_ 3359, 1748, 1705, 1602, 1516 cm^−1^; ^1^H-NMR (800 MHz) and ^13^C-NMR (200 MHz) data, see Table 2; HRMS (ESI-TOF) *m/z* [M + Na]^+^ 645.2146 (calcd. for C_30_H_38_O_14_Na, 645.2154).

*Cistansalside B* (**6**): brown amorphous powder; ${\left[\alpha \right]}_{D}^{20}$ −72.9 (*c* 0.1, MeOH); UV(MeOH) λ_max_ nm (log ε) 333 (3.32); IR (neat) ν_max_ 3400, 1705, 1603, 1516 cm^−1^; ^1^H-NMR (500 MHz) and ^13^C-NMR (125 MHz) data, see Table 2; HRMS (ESI-TOF) *m/z* [M + Na]^+^ 579.2054 (calcd. for C_26_H_36_O_13_Na, 579.2048).

*Cistansalside C* (**12**): brown amorphous powder; ${\left[\alpha \right]}_{D}^{20}$ −61.6 (*c* 0.1, MeOH); UV(MeOH) λ_max_ nm (log ε) 337 (3.25); IR (neat) ν_max_ 3359, 1704, 1602, 1508 cm^−1^; ^1^H-NMR (800 MHz) and ^13^C-NMR (200 MHz) data, see Table 2; HRMS (ESI-TOF) *m/z* [M + Na]^+^ 581.2213 (calcd. for C_26_H_38_O_13_Na, 581.2205).

*Cistansalside D* (**17**): brown amorphous powder; ${\left[\alpha \right]}_{D}^{20}$ −51.1 (*c* 0.1, MeOH); UV(MeOH) λ_max_ nm (log ε) 221 (3.53), 315 (3.52); IR (neat) ν_max_ 3358, 1746, 1722, 1603, 1516, 1232, 1157, 1039 cm^−1^; ^1^H-NMR (400 MHz) and ^13^C-NMR (75 MHz) data, see Table 2; HRMS (ESI-TOF) *m/z* [M + Na]^+^ 657.2147 (calcd. for C_31_H_38_O_14_Na, 657.2154).

*Cistansalside E* (**18**): brown amorphous powder; ${\left[\alpha \right]}_{D}^{20}$ −59.2 (*c* 0.1, MeOH); UV(MeOH) λ_max_ nm (log ε) 336 (3.22); IR (neat) ν_max_ 3370, 1741, 1712, 1602, 1231, 1157 cm^−1^; ^1^H-NMR (800 MHz) and ^13^C-NMR (200 MHz) data, see Table 2; HRMS (ESI-TOF) *m/z* [M + Na]^+^ 657.2166 (calcd. for C_31_H_38_O_14_Na, 657.2154).

### 3.5. HPLC-QTOF-MS Analysis

Chromatographic-mass spectrometry analysis was performed on an Agilent 1260 Infinity series LC system (Agilent Technologies, Inc., USA). The analytical column was a SFCpak Crest C18T-5 column (i.d. 150 × 4.6 mm, 5 μm, Jasco, Japan). The mobile phase consisted of 0.1% (*v/v*) formic acid in MeCN (A) and water (B) using a gradient elution of 0–35 min (23% A), 35–45 min (23–28% A), 45–75 min (28% A) and 75–80 min (90% A). The flow rate was kept at 0.3 mL/min. The absorbance was measured at 320 nm. The conditions of the ESI source were as follows: drying gas (N_2_) flow rate, 10 L/min; drying gas temperature, 350 °C; nebulizer, 30 psig; sheath gas flow rate, 12.0 L/min; sheath gas temperature, 350 °C; capillary, 4000 V; skimmer, 60 V; octapole RF, 750 V; fragmentor voltage, 180 V; positive mode. The system was operated under Masshunter workstation software. The mass range was set at *m/z* 50–1000.

### 3.6. Acid Hydrolysis

Compounds were hydrolyzed using 1 N H_2_SO_4_ (100 μL) heated with a water bath at 90 °C for 2 h, then neutralized with saturated aqueous Na_2_CO_3_ solution. After the solutions were dried under a stream of N_2_, the products and standard sugars (d-Glc, l-Glc, l-Rha) were dissolved in pyridine (100 μL) containing l-cysteine methyl ester hydrochloride (0.5 mg). An l-rhamnose sample was dissolved in pyridine (100 μL) containing d-cysteine methyl ester hydrochloride (0.5 mg). After that, they were heated at 60 °C for 1 h. The solutions were treated with 1 μL (1.11 mg) of *o*-tolylisothiocyanate, which were heated again at 60 °C for 1 h. Each final mixture was directly analyzed by analytical RP-HPLC (Hypersil™ BDS C18 column, 17% aq. MeCN, 0.8 mL/min, 40 min, 35 °C). The t_R_ of the peak at 21.9 and 40.4 min coincided with that of the thiocarbamoyl thiazolidine derivative of d-glucose and l-rhamnose, respectively.

### 3.7. Cell Culture

Murine macrophages, RAW 264.7, were obtained from the Korean Research Institute of Bioscience and Biotechnology (Daejeon, Korea), and grown in RPMI medium containing 10% fetal bovine serum and 100 U/mL penicillin/streptomycin sulfate. Cells were incubated in a humidified 5% CO_2_ atmosphere at 37 °C.

### 3.8. Drugs and Chemicals

RPMI, penicillin and streptomycin were purchased from HyClone (Logan, UT, USA). Bovine serum albumin and LPS were purchased from Sigma (St. Louis, MO, USA). 

### 3.9. Measurement of NO Production

The nitrite concentration in the culture medium was measured as an indicator of NO production according to the Griess reaction. The cells were seeded at 2 × 10^5^ cells/well in 96-well culture plates. After pre-incubation of the RAW 264.7 cells for 18 h, the cells were pretreated with compounds (50 µM, 10 µM, 5 µM or 1 µM) and stimulated with LPS (500 ng/mL) for 24 h. Test compounds dissolved in DMSO. Cells were also treated with 0.05% DMSO as a vehicle control. RAW 264.7 cells (2 × 10^5^ cells/well) were cultured in 96-well plates using RPMI without phenol red, and pretreated with samples for 0.5 h. Cellular NO production was induced by the addition of 500 ng/mL final concentration LPS and a 24 h incubation. Following incubation, 100 µL of conditioned media was mixed with the same volume of Griess reagent and incubated for 15 min. The absorbance of the mixture at 540 nm was measured with an ELISA microplate reader (Benchmark, Bio-Rad Laboratories, Richmond, CA, USA). The values obtained were compared with those of standard concentrations of sodium nitrite dissolved in RPMI, and the concentrations of nitrite in the conditioned media of sample-treated cells were calculated.

### 3.10. 3-(4,5-Dimethylthiazol-2-yl)-2,5-diphenyltetrazolium Bromide (MTT) Assay for Cell Viability

Cells were seeded into 96-well plates at a density of 5 × 10^4^ cells/well and incubated with serum-free media in the presence of samples. Test compounds dissolved in DMSO. Cells were also treated with 0.05% DMSO as a vehicle control. Following incubation for 24 h, 10 µL MTT (5 mg/mL in saline) was added and incubation was continued for further 4 h. Mitochondrial succinate dehydrogenase in live cells converts MTT into visible formazan crystals during incubation. The formazan crystals were then solubilized in dimethyl sulfoxide and the absorbance was measured at 540 nm using an enzyme-linked immunosorbent assay (ELISA) microplate reader (Benchmark, Bio-Rad Laboratories). Relative cell viability was calculated compared with the absorbance of the untreated control group. All experiments were performed in triplicate.

### 3.11. Immunoblot Analysis

Protein expression was assessed by western blotting according to standard procedures. Briefly, RAW264.7 cells were cultured in 60 mm culture dishes (2 × 10^6^/mL), following by pretreatment 50 μM of compounds. Cells were washed twice in ice cold PBS (pH 7.4), the cell pellets were resuspended in lysis buffer on ice for 15 min, and the cell debris was then removed by centrifugation. Protein concentration was determined using Bio-Rad protein assay reagent according to the manufacturer’s instructions. Protein (20–30 μg) was mixed 1:1 with 2× sample buffer (20% glycerol, 4% SDS, 10% 2-ME, 0.05% bromophenol blue, and 1.25 M Tris [pH 6.8]), loaded onto 8 or 15% SDS-PAGE gels, and run at 150 V for 90 min. Cellular proteins were transferred onto ImmunoBlot polyvinylidene difluoride membranes (Bio-Rad) using a Bio-Rad semi-dry transfer system according to the manufacturer’s instructions. The membranes were then incubated overnight with the resprective p-NF-κB, NF-κB, p-IκBα and β-actin primary antibodies (Abcam, Cambridge, UK) in Tris-buffered saline containing 5% skimmed milk and 0.1% Tween 20. The following day, the blots were washed three times with Tris-buffered saline (0.1% Tween 20) and incubated for 1 h with an HRP-conjugated secondary anti-IgG antibody (diluted 1:2000–1:20,000). The blots were washed again three times with Tris-buffered saline (0.1% Tween 20), and immunoreactive bands were developed using the chemiluminescent substrate ECL Plus (Amersham Biosciences, Piscataway, NJ, USA).

### 3.12. Statistical Analysis

Experimental data are presented as the mean ± SEM. The level of statistical significance was determined by analysis of variance (ANOVA) followed by Dunnett’s *t*-test for multiple comparisons. *p* Values less than 0.05 were considered significant.

## 4. Conclusions

In this study, we isolated and elucidated the structures of five new phenylpropanoid-substituted diglycosides, named cistansalside A-E (**5**, **6**, **12**, **17** and **18)**, in addition to isolating and identifying 13 known compounds, using a dereplication strategy. The known compounds were determined to be lipedoside A-I (**1**) [21], 2′-acetylacteoside (**2**) [22], isocistanoside C (**3**) [15], osmanthuside B (**4**) [21], epimeridinoside A (**7**) [15], cistanoside D (**8**) [23], salsaside B (**9**) [6], tubuloside E (**10**) [16], cistanoside M (**11**) [15], isomartynoside (**13**) [24], salsaside C (**14**) [6], jionoside C (**15**) [25] and salsaside F (**16**) [6]. Their structures were established through the analysis of extensive spectroscopic data and by comparison to reported data in the literature. It was confirmed that tentatively predicted structures of phenylpropanoid-substituted diglycosides were correctly matched to their real structures. 

In NO inhibitory assay, compounds **5** (IC_50_ 42.7 ± 6.6 μM), **11** (IC_50_ 37.3 ± 2.2 μM), **13** (IC_50_ 40.0 ± 4.0 μM) and **18** (IC_50_ 27.9 ± 0.8 μM) showed moderate inhibitory activities. Of these compounds, compounds **11**, **13** and **18** were found to inhibit the phosphorylation of NF-κB in macrophages and might thus exert an anti-inflammatory activity which will have to be proven in further experiments.

## Figures and Tables

**Table 1:** Identification of phenylpropanoid-substituted diglycosides in EtOAc fraction of *C. salsa* by HPLC-ESI-QTOF-MS in positive ion mode.

No.	t_R_	[M + Na]^+^	M.W.	Molecular Formula	MS Fragment Ions	Fragment ^c^	Identification	Abundances ^d^ (%)
X1 ^b^	11.094	647.1954	624	C_29_H_36_O_15_	163, 325, 471, 642, 647, 663	Caf, Glc, Rha, D	-	-
X2 ^b^	13.939	647.1920	624	C_29_H_36_O_15_	163, 325, 471, 642, 647, 663	Caf, Glc, Rha, D	-	-
X3 ^b^	15.701	631.1959	608	C_29_H_36_O_14_	163, 325, 471, 626, 631, 647	Caf, Glc, Rha, C	-	-
1	16.170	631.1982	608	C_29_H_36_O_14_	147, 309, 455, 605, 626, 631, 647	Cou, Glc, Rha, D	Lipedoside A-I	1.9
X4 ^b^	16.962	661.2103	638	C_30_H_38_O_15_	163, 325, 471, 656, 661, 679	Caf, Glc, Rha, E or F	-	-
2	20.858	689.2070	666	C_31_H_38_O_16_	163, 367, 513, 684, 689, 705	Caf, Acetyl-Glc, Rha, D	2′-Acetylacteoside	50.0 (8.5)
3	22.288	661.2116	638	C_30_H_38_O_15_	163, 325, 471, 656, 661, 679	Caf, Glc, Rha, F	Isocistanoside C	1.3
4	24.640	615.2019	592	C_29_H_36_O_13_	147, 309, 455, 593, 610, 615, 631	Cou, Glc, Rha, C	Osmanthuside B	1.4
5 ^a^	26.645	645.2146	622	C_30_H_38_O_14_	177, 339, 485, 640, 645, 661	Fer, Glc, Rha, C	Cistansalside A	<1.0
6 ^a^	26.696	579.2054	556	C_26_H_36_O_13_	163, 325, 471, 574, 579, 595	Caf, Glc, Rha, G	Cistansalside B	<1.0
X5 ^b^	27.744	689.2027	666	C_31_H_38_O_16_	163, 367, 513, 684, 689, 705	Caf, Acetyl-Glc, Rha, D	-	-
7	28.901	675.2248	652	C_31_H_40_O_15_	177, 339, 485, 653, 670, 675, 691	Fer, Glc, Rha, F	Epimeridinoside A	<1.0
8	28.989	675.2247	652	C_31_H_40_O_15_	177, 339, 485, 653, 670, 675, 691	Fer, Glc, Rha, F	Cistanoside D	<1.0
X6 ^b^	31.963	689.2027	666	C_31_H_38_O_16_	163, 367, 513, 684, 689, 705	Caf, Acetyl-Glc, Rha, D	-	-
9	32.824	601.1890	578	C_28_H_34_O_13_	163, 325, 471, 579, 601, 617	Caf, Glc, Rha, A	Salsaside B	4.8 (<1.0)
10	34.420	673.2136	650	C_31_H_38_O_15_	147, 351, 497, 668, 673, 689	Cou, Acetyl-Glc, Rha, D	Tubuloside E	5.3 (1.2)
11	40.315	645.2168	622	C_30_H_38_O_14_	147, 309, 455, 640, 645, 661	Cou, Glc, Rha, F	Cistanoside M	<1.0
12 ^a^	41.954	581.2213	558	C_26_H_38_O_13_	163, 325, 471, 576, 581, 597	Caf, Glc, Rha, I	Cistansalside C	<1.0
13	44.636	675.2248	652	C_31_H_40_O_15_	177, 339, 485, 653, 670, 675, 691	Fer, Glc, Rha, E	Isomartynoside	<1.0
14	50.569	585.2004	562	C_28_H_34_O_12_	147, 309, 455, 563, 580, 585, 601	Cou, Glc, Rha, A	Salsaside C	<1.0
15	51.199	615.2082	592	C_29_H_36_O_13_	163, 325, 471, 593, 610, 615, 631	Caf, Glc, Rha, B	Jionoside C	<1.0
16	51.878	673.2137	650	C_31_H_38_O_15_	147, 351, 497, 668, 673, 689	Cou, Acetyl-Glc, Rha, D	Salsaside F	<1.0
17 ^a^	53.672	657.2147	634	C_31_H_38_O_14_	147, 351, 497, 652, 657, 673	Cou, Acetyl-Glc, Rha, C	Cistansalside D	<1.0
18 ^a^	81.127	657.2166	634	C_31_H_38_O_14_	163, 367, 513, 652, 657, 673	Caf, Acetyl-Glc, Rha, B	Cistansalside E	<1.0

^a^ new compounds. ^b^ X1–6 have not been identified yet. ^c^ Aglycone substituents A-I were shown in Figure 1. ^d^ Abundances in the EtOAc fraction were measured by LC-PDA (320 nm). Abundances in crude extract were in parenthesis.

**Table 2:** ^1^H and ^13^C-NMR Data of new compounds (DMSO-*d_6_*).

	5 ^a^		6 ^b^		12 ^a^		17 ^c^		18 ^a^	
Position	δ_C_	δ_H_ (*J* in Hz)	δ_C_	δ_H_ (*J* in Hz)	δ_C_	δ_H_ (*J* in Hz)	δ_C_	δ_H_ (*J* in Hz)	δ_C_	δ_H_ (*J* in Hz)
1	128.5		64.5	4.23 dd (6.5, 12.2)	67.2	3.81 m	128.6		138.8	
				4.13 dd (7.6, 12.2)		3.48 m				
2	129.8	7.05 d (8.3)	120.7	5.13 dd (7.6, 6.5)	38.0	1.44 m	129.7	6.98 d (8.0)	128.9	7.21 m
3	115.0	6.67 d (8.3)	136.4		24.4	1.71 m	114.9	6.65 d (8.0)	128.2	7.29 m
4	155.7		25.5	1.71 s	22.5	0.88 d (6.7)	155.6		126.0	7.20 m
5	115.0	6.67 d (8.3)	17.8	1.63 s	22.5	0.88 d (6.7)	114.9	6.65 d (8.0)	128.2	7.29 m
6	129.8	7.05 d (8.3)					129.7	6.98 d (8.0)	128.9	7.21 m
7	34.7	2.76 m					34.4	2.66 t (6.1)	35.2	2.80 m
8	70.2	3.90 m					69.8	3.90 m	69.4	3.99 m
		3.61 m						3.54 m		3.63 m
1′	102.3	4.35 d (7.9)	100.9	4.29 d (7.9)	102.3	4.28 d (7.9)	99.2	4.61 m	99.2	4.63 d (8.2)
2′	74.4	3.21 m	74.5	3.18 m	74.5	3.18 m	73.4	4.69 m	73.5	4.71 dd (8.9, 8.2)
3′	79.2	3.68 m	79.0	3.68 m	79.1	3.68 m	78.0	3.95 dd (9.3, 9.3)	77.9	3.95 m
4′	69.1	4.71 dd (9.8, 9.8)	69.2	4.70 dd (9.6, 9.6)	69.2	4.70 dd (9.4, 9.4)	68.9	4.80 m	69.0	4.81 dd (9.7, 9.5)
5′	74.5	3.44 m	74.5	3.44 m	74.5	3.43 m	74.4	3.56 m	74.5	3.58 m
6′	60.7	3.40 m	60.8	3.38 m	60.8	3.38 m	60.4	3.41 m	60.5	3.40 m
		3.34 m		3.34 m		3.28 m		3.34 m		3.36 m
1″	101.3	5.02 s	101.2	5.01 s	101.2	5.01 s	102.0	4.60 s	102.0	4.60 s
2″	70.5	3.68 m	70.5	3.66 m	70.5	3.66 m	70.7	3.39 m	70.7	3.38 m
3″	70.4	3.27 m	70.4	3.27 m	70.4	3.27 m	70.1	3.22 m	70.1	3.22 m
4″	71.6	3.11 m	71.6	3.10 m	71.6	3.10 m	71.4	3.08 m	71.4	3.08 m
5″	68.8	3.36 m	68.7	3.34 m	68.7	3.34 m	69.2	3.29 m	69.3	3.27 m
6″	18.1	0.97 d (6.3)	18.1	0.95 d (6.1)	18.2	0.95 d (6.1)	18.0	0.92 d (6.1)	18.2	0.92 d (6.1)
1′″	125.6		125.5		125.5		124.9		125.4	
2′″	111.1	7.29 d (1.3)	114.7	7.02 s	114.7	7.02 d (1.3)	115.8	6.79 d (6.9)	114.8	7.02 d (1.6)
3′″	147.9		148.5		148.5		130.3	7.53 d (6.9)	145.7	
4′″	149.4		149.0		149.3		160.0		148.7	
5′″	115.5	6.79 d (8.3)	115.8	6.75 d (8.3)	115.8	6.76 d (8.1)	130.3	7.53 d (6.9)	115.8	6.76 d (8.1)
6′″	123.2	7.09 dd (8.3, 1.3)	121.5	6.97 d (8.3)	121.4	6.97 dd (8.1, 1.3)	115.8	6.79 d (6.9)	121.5	6.98 dd (8.1, 1.6)
7′″	145.5	7.53 d (15.9)	145.6	7.47 d (15.8)	145.6	7.45 d (15.5)	145.4	7.55 d (15.9)	145.5	7.48 d (15.9)
8′″	114.1	6.41 d (15.9)	113.5	6.19 d (15.8)	113.6	6.19 d (15.5)	113.5	6.34 d (15.9)	113.2	6.21 d (15.9)
9′″	165.8		165.7		165.7		165.5		165.6	
3′″-OCH_3_	55.6	3.80 s								
Acetyl-CO							169.0		169.1	
Acetyl-CH_3_							20.5	1.97 s	20.6	1.94 s

^a 1^H and ^13^C-NMR spectra for **5**, **12** and **18** were obtained with a Bruker Avance 800 HD spectrometer (Bruker, Ettlingen, Germany) coupled with a cryoprobe. ^b 1^H and ^13^C-NMR spectra for **6** were obtained with a Bruker Avance-500 (Bruker, Ettlingen, Germany). ^c 1^H and ^13^C-NMR spectra for **17** were obtained with a Jeol LA 300 (Jeol, Tokyo, Japan).

## Supplementary Materials

Supplementary data associated with this article can be found in the PDF file (supplementary material).
