# Supplementary material for: Dereplication-Guided Isolation of New Phenylpropanoid-Substituted Diglycosides from Cistanche salsa and Their Inhibitory Activity on NO Production in Macrophage
Source: Molecules. 2017 Jul 8;22(7):1138. doi: 10.3390/molecules22071138 (PMC6152099; doi:10.3390/molecules22071138)

SUPPORTING INFORMATION

Dereplication-guided Isolation of New Phenylpropanoid-substituted Diglycosides from *Cistanche salsa* and Their Inhibitory Activity on NO Production in Macrophage

Jongmin Ahn [1], Hee-Sung Chae [2], Young-Won Chin [2] and Jinwoong Kim [1,*]

[1] College of Pharmacy and Research Institute of Pharmaceutical Sciences, Seoul National University, Seoul 08826, Republic of Korea

[2] College of Pharmacy and Integrated Research Institute for Drug Development, Dongguk University-Seoul, Gyeonggi-do 10326, Republic of Korea

* Correspondence: jwkim@snu.ac.kr (J. Kim); Tel.: +82-2-880-7853

List of Supplementary material :

No.	Content	Page
**Figure S1-1**	The HRESIMS of **5.**	3
**Figure S1-2**	The ^{1}H NMR (800 MHz) spectrum of **5** in DMSO-d_6	4
**Figure S1-3**	The ^{13}C NMR (200 MHz) spectrum of **5** in DMSO-d_6	5
**Figure S1-4**	The HSQC spectrum of **5** in DMSO-d_6	6
**Figure S1-5**	The ^{1}H-^{1}H COSY spectrum of **5** in DMSO-d_6	7
**Figure S1-6**	The HMBC spectrum of **5** in DMSO-d_6	8
**Figure S1-7**	The ^{1}H-^{1}H NOESY spectrum of **5** in DMSO-d_6	9
**Figure S1-8**	The UV spectrum of **5**	10
**Figure S2-1**	The HRESIMS of **6.**	11
**Figure S2-2**	The ^{1}H NMR (500 MHz) spectrum of **6** in DMSO-d_6	12
**Figure S2-3**	The ^{13}C NMR (125 MHz) spectrum of **6** in DMSO-d_6	13
**Figure S2-4**	The HSQC spectrum of **6** in DMSO-d_6	14
**Figure S2-5**	The ^{1}H-^{1}H COSY spectrum of **6** in DMSO-d_6	15
**Figure S2-6**	The HMBC spectrum of **6** in DMSO-d_6	16
**Figure S2-7**	The UV spectrum of **6**	17
**Figure S3-1**	The HRESIMS of **12.**	18
**Figure S3-2**	The ^{1}H NMR (800 MHz) spectrum of **12** in DMSO-d_6	19
**Figure S3-3**	The ^{13}C NMR (200 MHz) spectrum of **12** in DMSO-d_6	20
**Figure S3-4**	The HSQC spectrum of **12** in DMSO-d_6	21
**Figure S3-5**	The ^{1}H-^{1}H COSY spectrum of **12** in DMSO-d_6	22
**Figure S3-6**	The HMBC spectrum of **12** in DMSO-d_6	23
**Figure S3-7**	The UV spectrum of **12**	24
**Figure S4-1**	The HRESIMS of **17.**	25
**Figure S4-2**	The ^{1}H NMR (300 MHz) spectrum of **17** in DMSO-d_6	26
**Figure S4-3**	The ^{13}C NMR (75 MHz) spectrum of **17** in DMSO-d_6	27
**Figure S4-4**	The HSQC spectrum of **17** in DMSO-d_6	28
**Figure S4-5**	The ^{1}H-^{1}H COSY spectrum of **17** in DMSO-d_6	29
**Figure S4-6**	The HMBC spectrum of **17** in DMSO-d_6	30
**Figure S4-7**	The UV spectrum of **17**	31
**Figure S5-1**	The HRESIMS of **18.**	32
**Figure S5-2**	The ^{1}H NMR (800 MHz) spectrum of **18** in DMSO-d_6	33
**Figure S5-3**	The ^{13}C NMR (200 MHz) spectrum of **18** in DMSO-d_6	34
**Figure S5-4**	The HSQC spectrum of **18** in DMSO-d_6	35
**Figure S5-5**	The ^{1}H-^{1}H COSY spectrum of **18** in DMSO-d_6	36
**Figure S5-6**	The HMBC spectrum of **18** in DMSO-d_6	37
**Figure S5-7**	The UV spectrum of **18**	38
**Figure S6-1**	Effects of compounds on the NO production and cell viability of RAW264.7 cells.	39
**Figure S6-2**	Dose-response curves of compounds **5**, **11**, **13** and **18**	40

Figure S 1- 1. The HRESIMS of **5.**

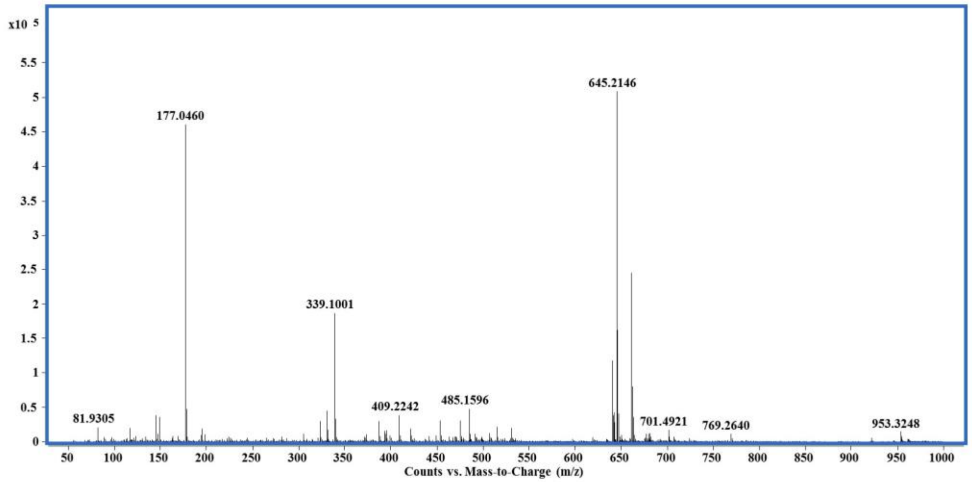

Formula (M)	Ion Formula	Mass (MFG)	m/z (Calc)	Diff (ppm)
C28 H39 Na O14	C28 H39 Na2 O14	622.2238	645.213	-2.62
C30 H38 O14	C30 H38 Na O14	622.2262	645.2154	1.25
C46 H31 Na O	C46 H31 Na2 O	622.2273	645.2165	3.02
C21 H43 Na O19	C21 H43 Na2 O19	622.2296	645.2188	6.82
C48 H30 O	C48 H30 Na O	622.2297	645.2189	6.89

Figure S 1- 2. The ^{1}H NMR (800 MHz) spectrum of **5** in DMSO-d_6

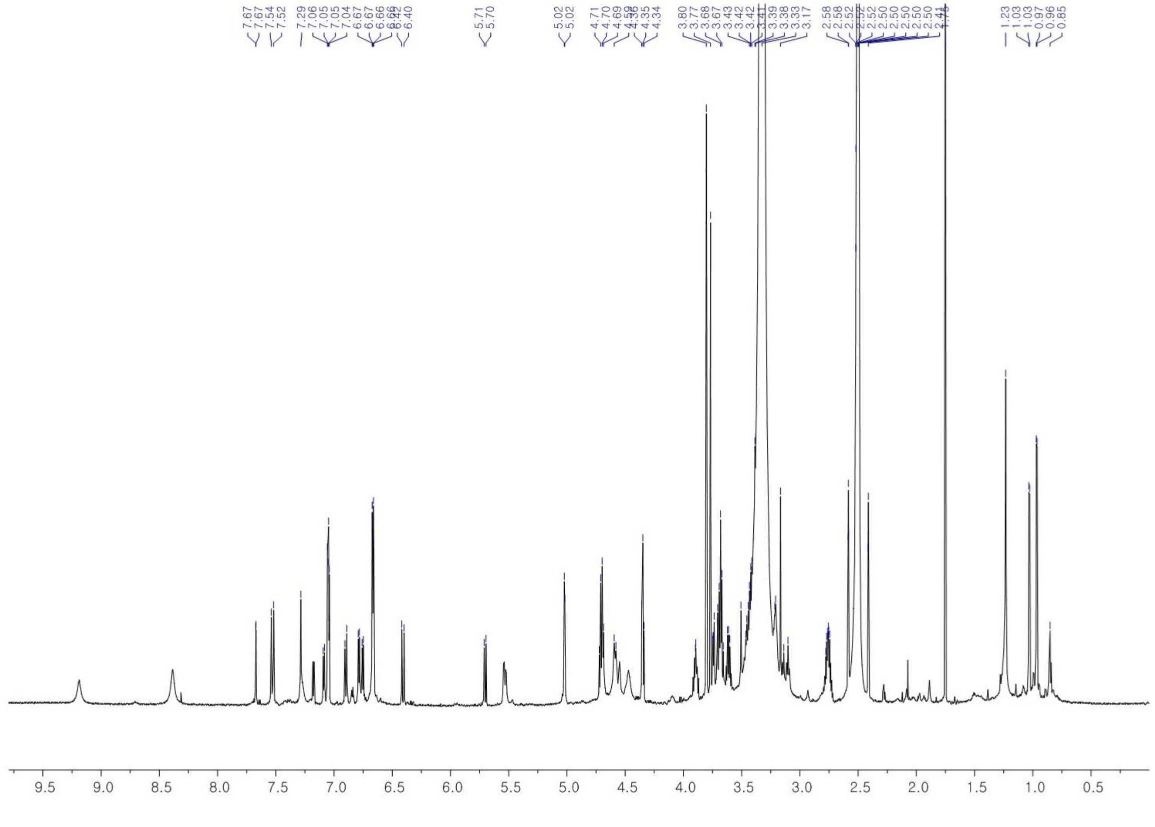

Figure S 1- 3 The ^{13}C NMR (200 MHz) spectrum of **5** in DMSO-d_6

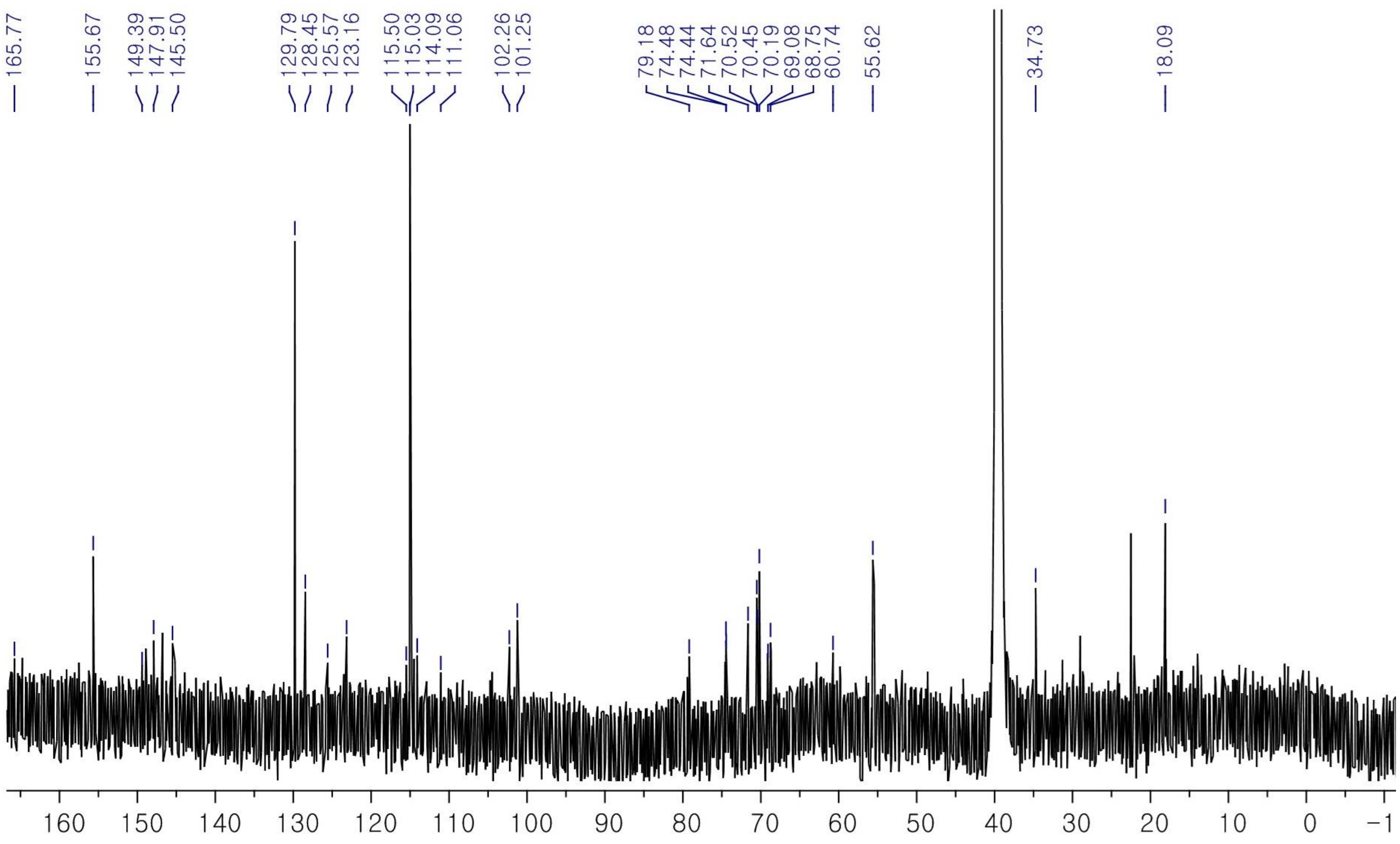

Figure S 1- 4. The HSQC spectrum of **5** in DMSO-d_6

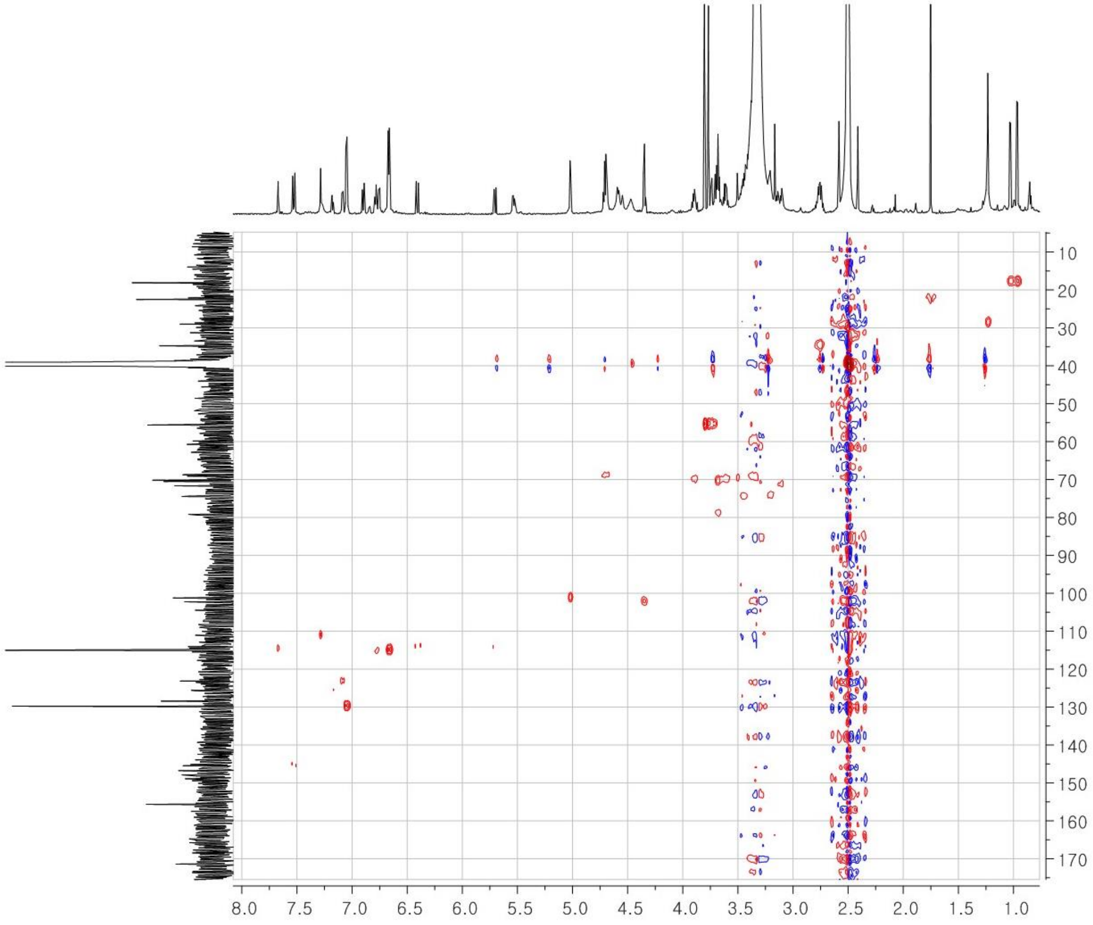

Figure S 1- 5. The COSY spectrum of **5** in DMSO-d_6

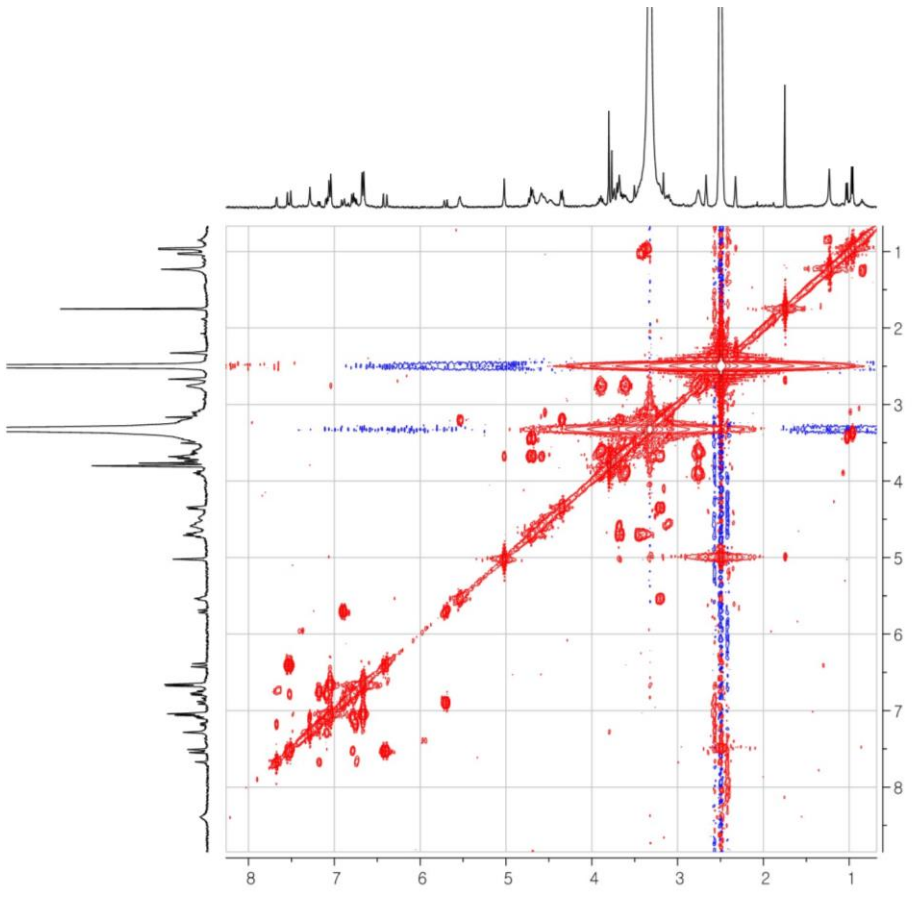

Figure S 1- 6. The HMBC spectrum of **5** in DMSO-d_6

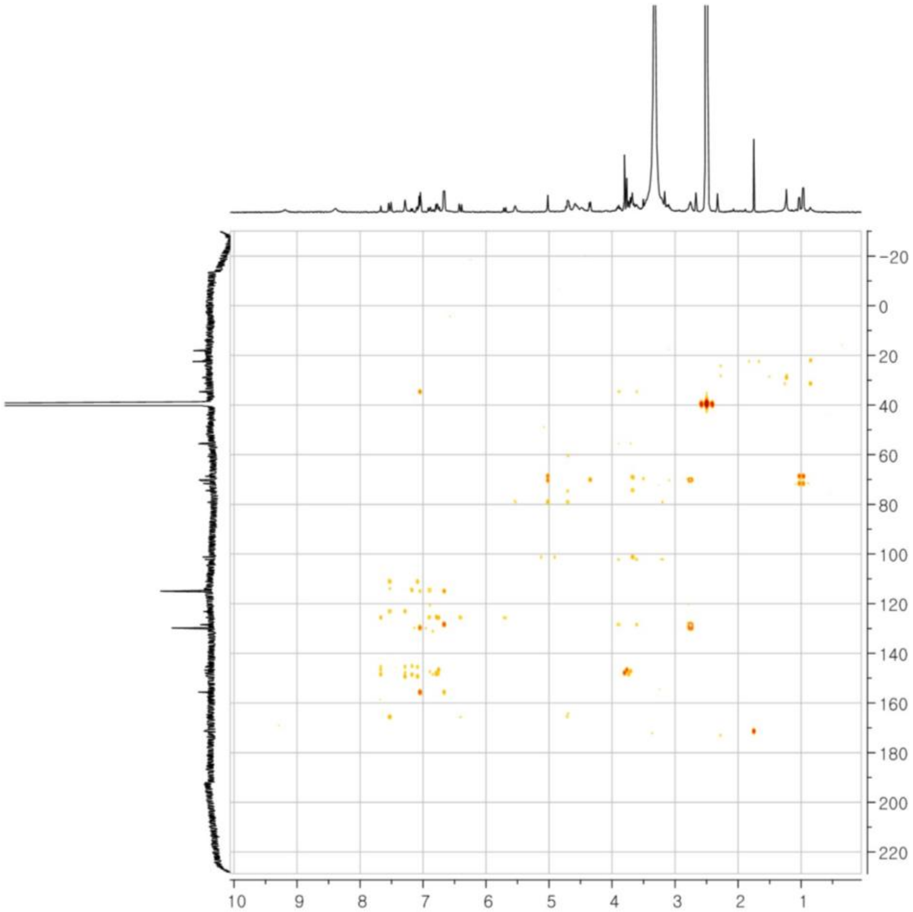

Figure S 1- 7. The NOESY spectrum of **5** in DMSO-d_6

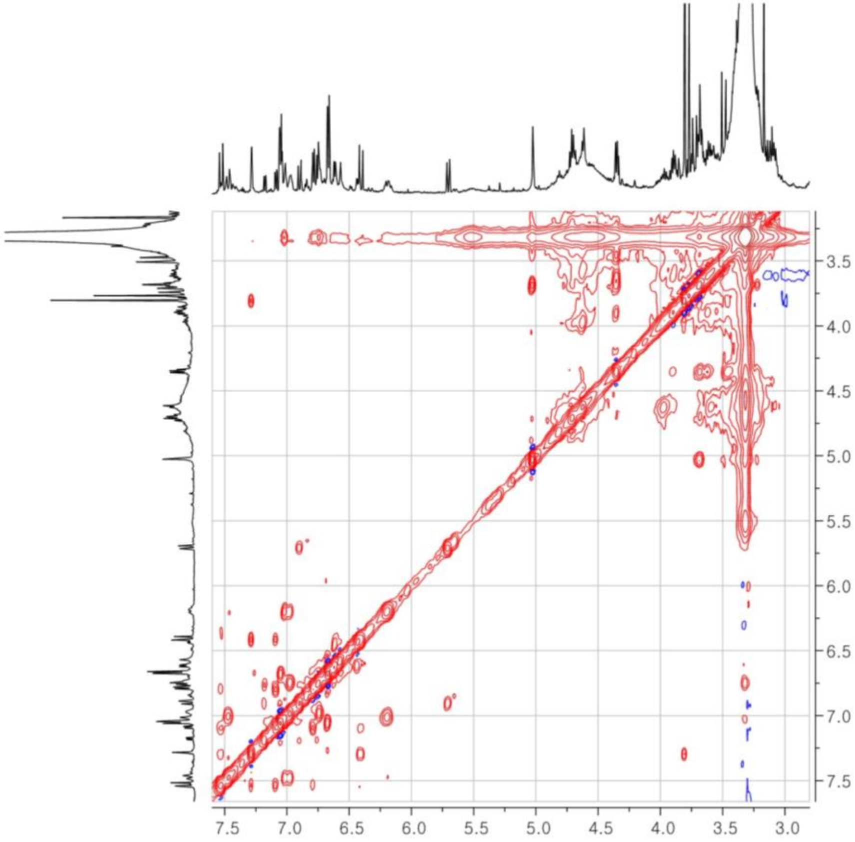

Figure S 1- 8. The UV spectrum of **5**

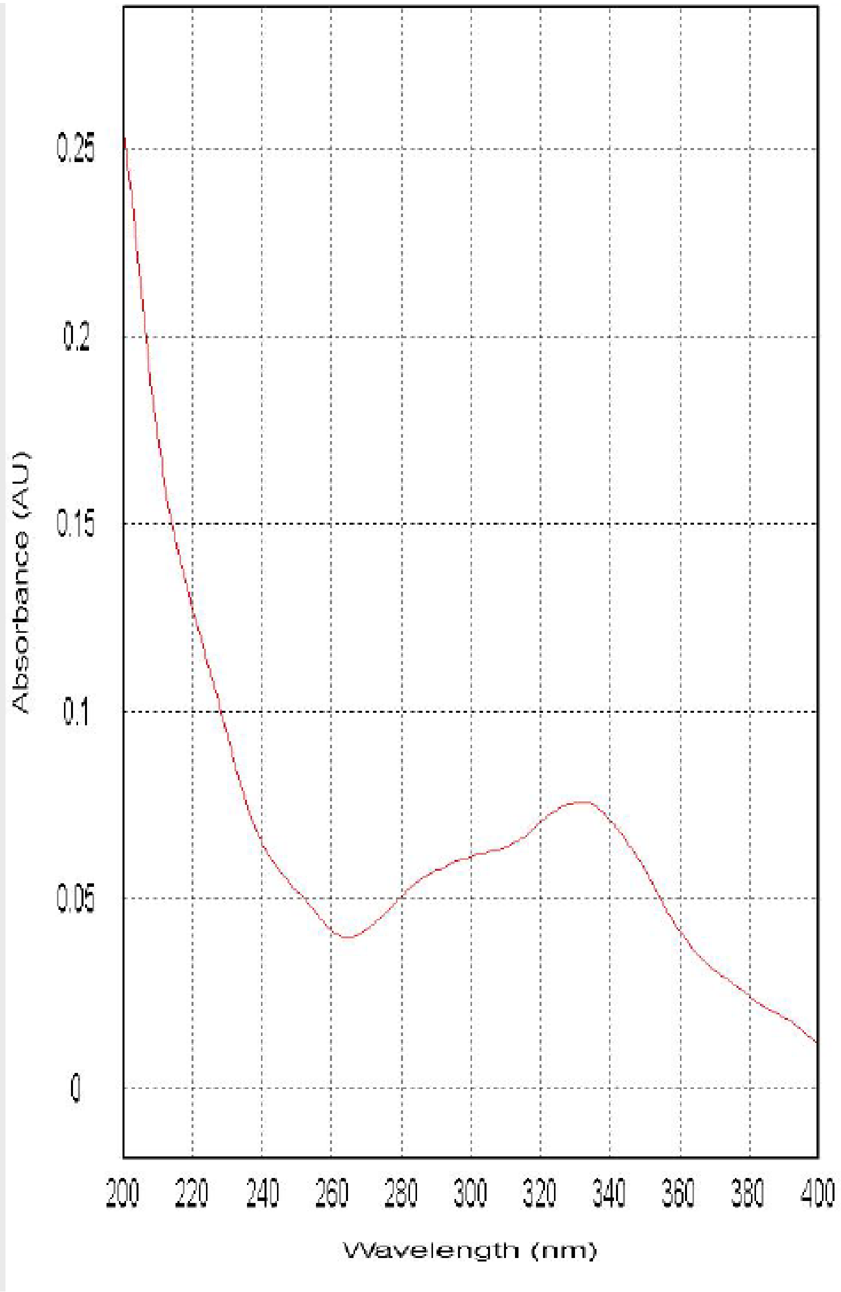

Figure S 2- 1 The HRESIMS of **6.**

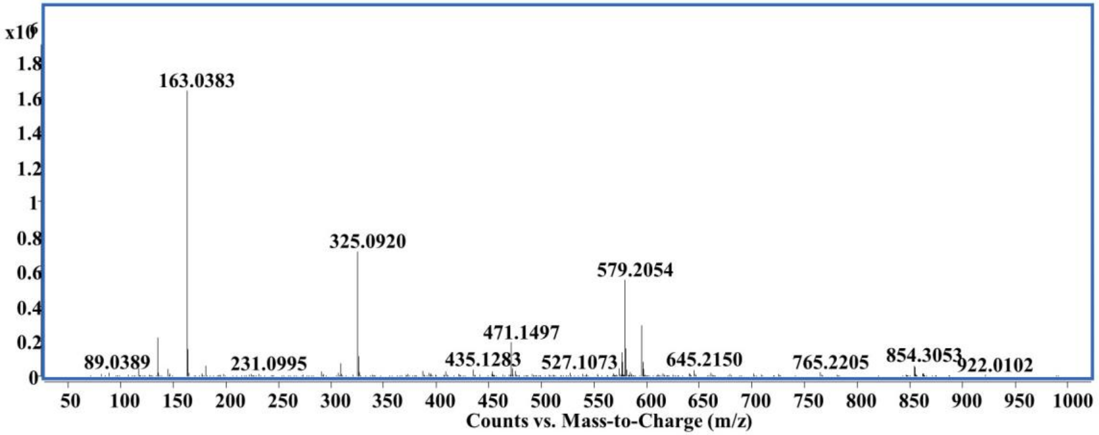

Formula (M)	Ion Formula	Mass (MFG)	m/z (Calc)	Diff (ppm)
C24 H37 Na O13	C24 H37 Na2 O13	556.2132	579.2024	-5.38
C26 H36 O13	C26 H36 Na O13	556.2156	579.2048	-1.06
C42 H29 Na	C42 H29 Na2	556.2167	579.2059	0.93
C44 H28	C44 H28 Na	556.2191	579.2083	5.25

Figure S 2- 2. The ^{1}H NMR (500 MHz) spectrum of **6** in DMSO-d_6

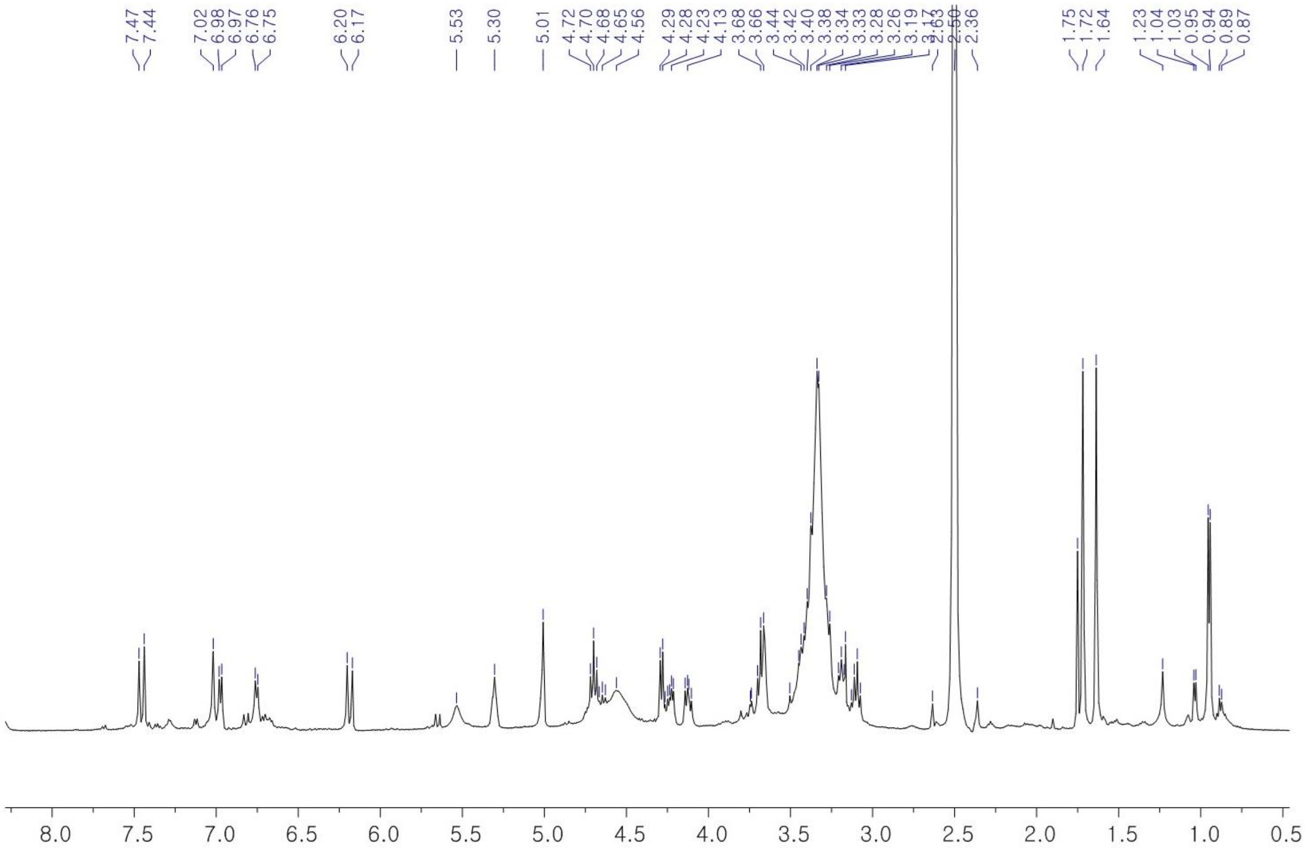

Figure S 2- 3. The ^{13}C NMR (125 MHz) spectrum of **6** in DMSO-d_6

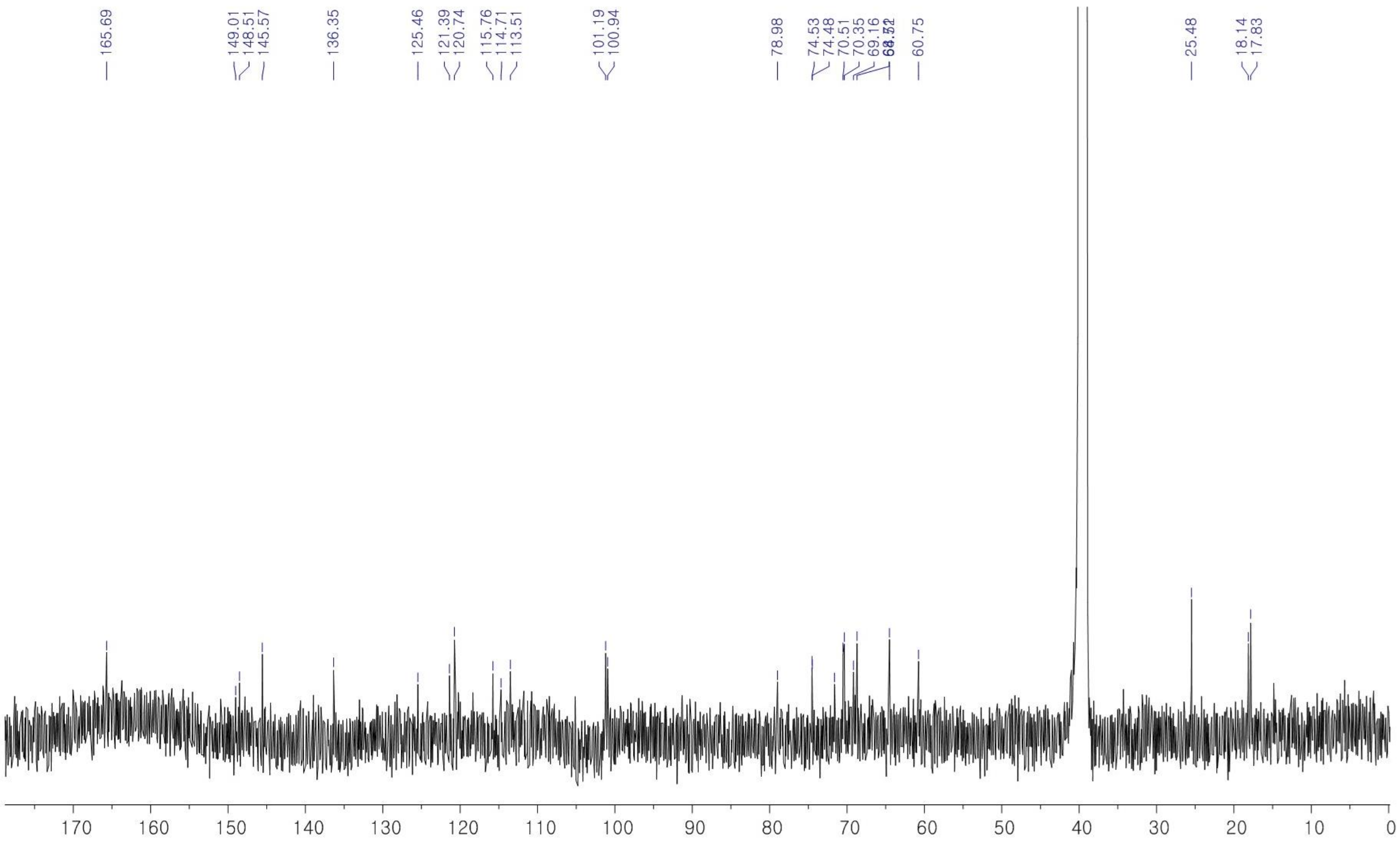

Figure S 2- 4. The HSQC spectrum of **6** in DMSO-d_6

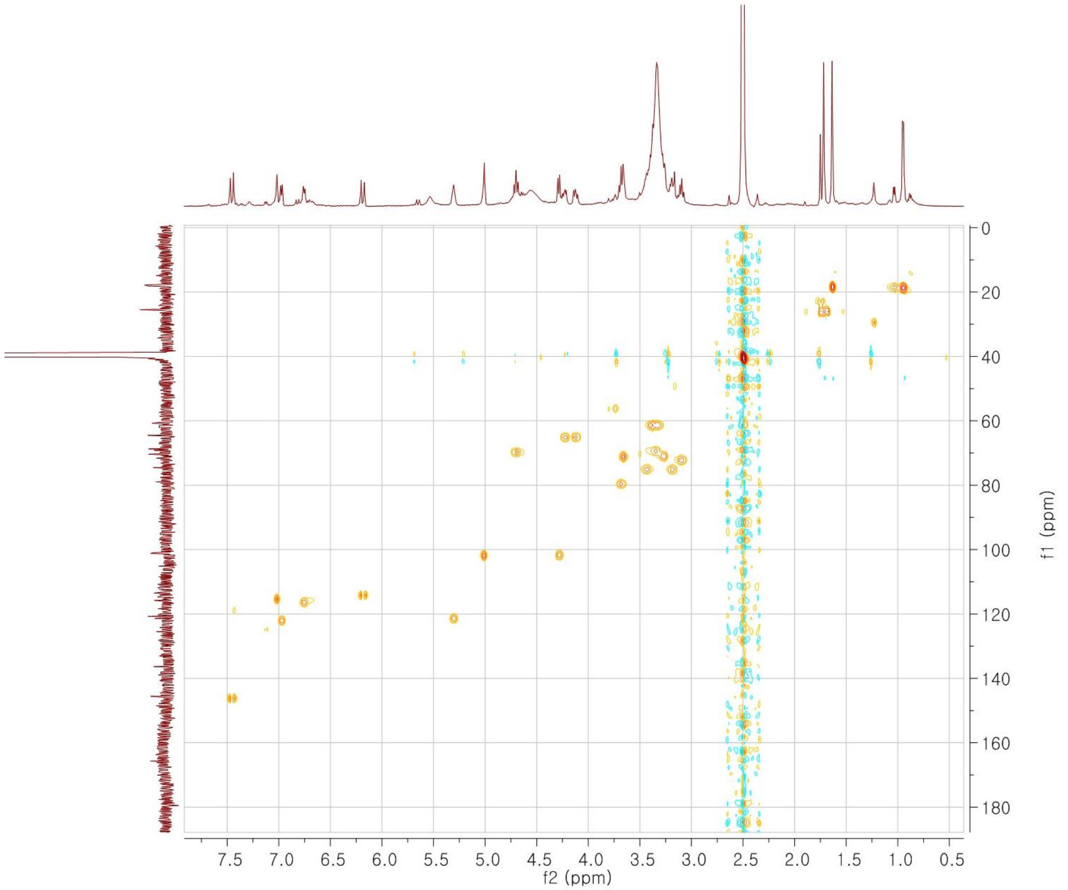

Figure S 2- 5. The COSY spectrum of **6** in DMSO-d_6

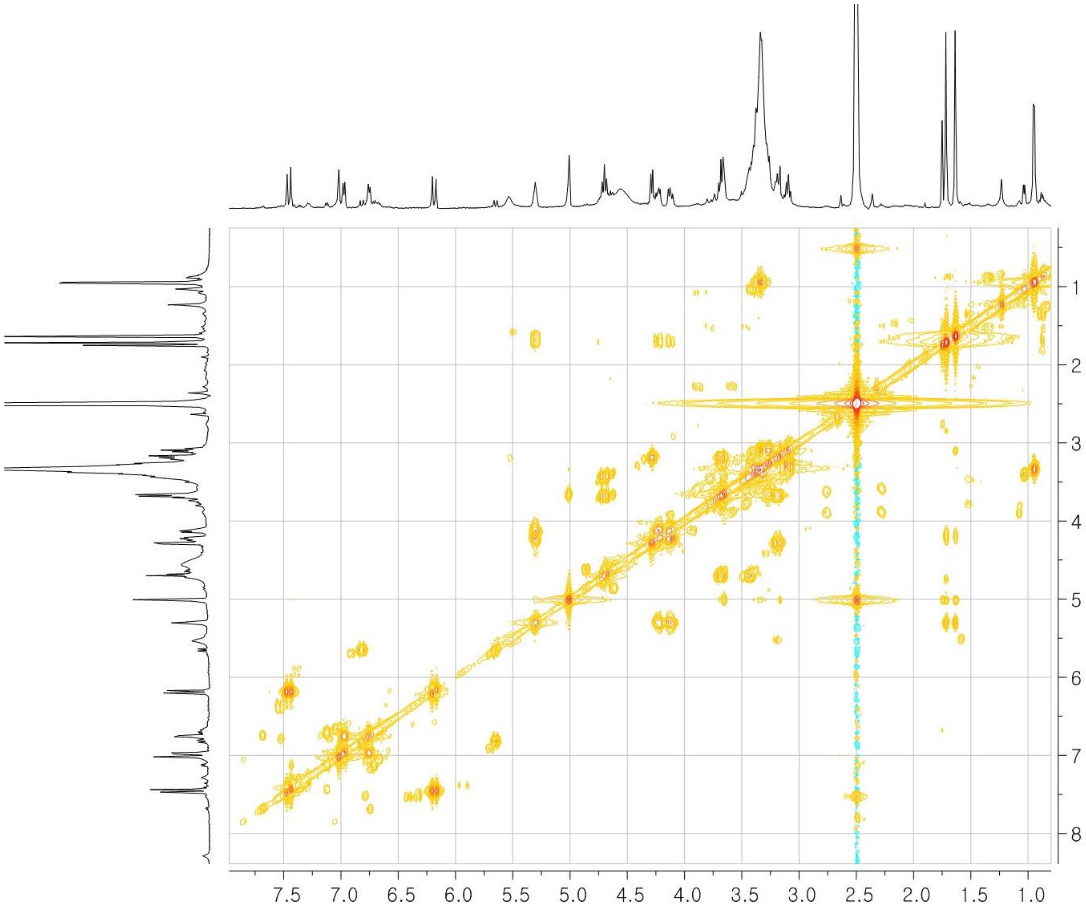

Figure S 2- 6. The HMBC spectrum of **6** in DMSO-d_6

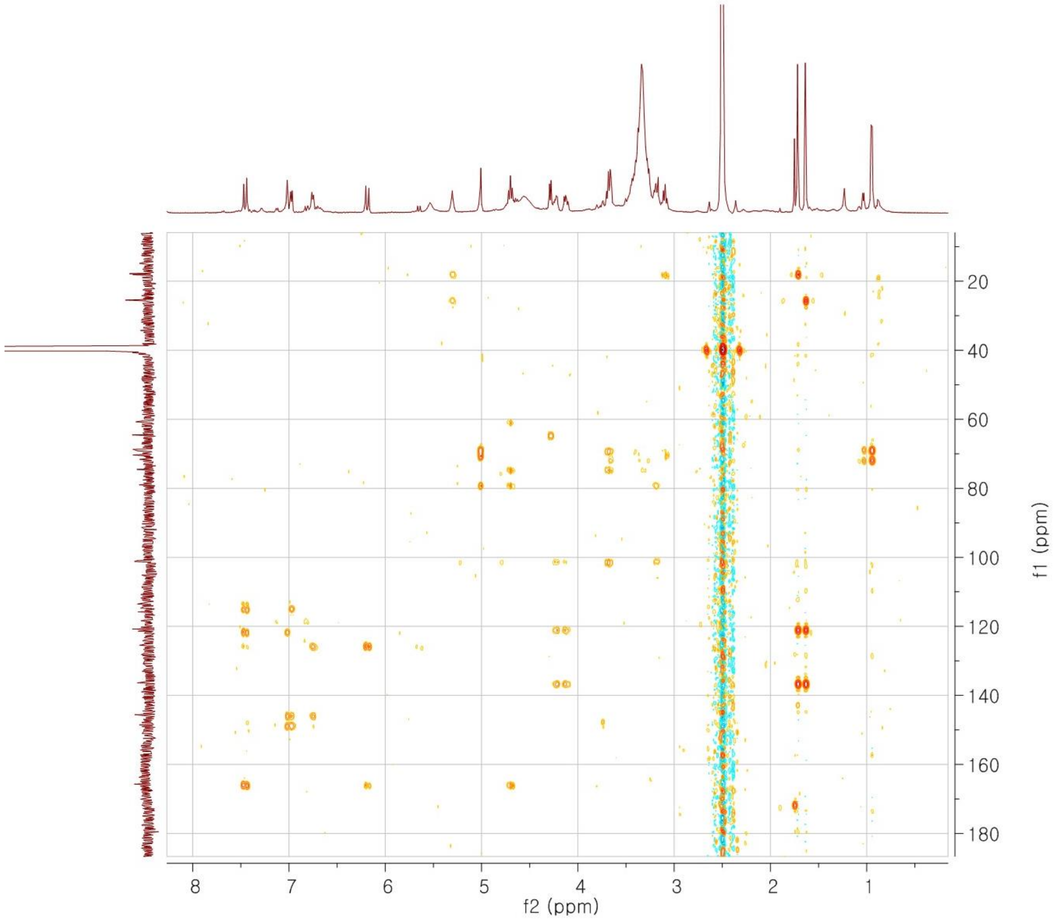

Figure S 2- 7. The UV spectrum of **6**

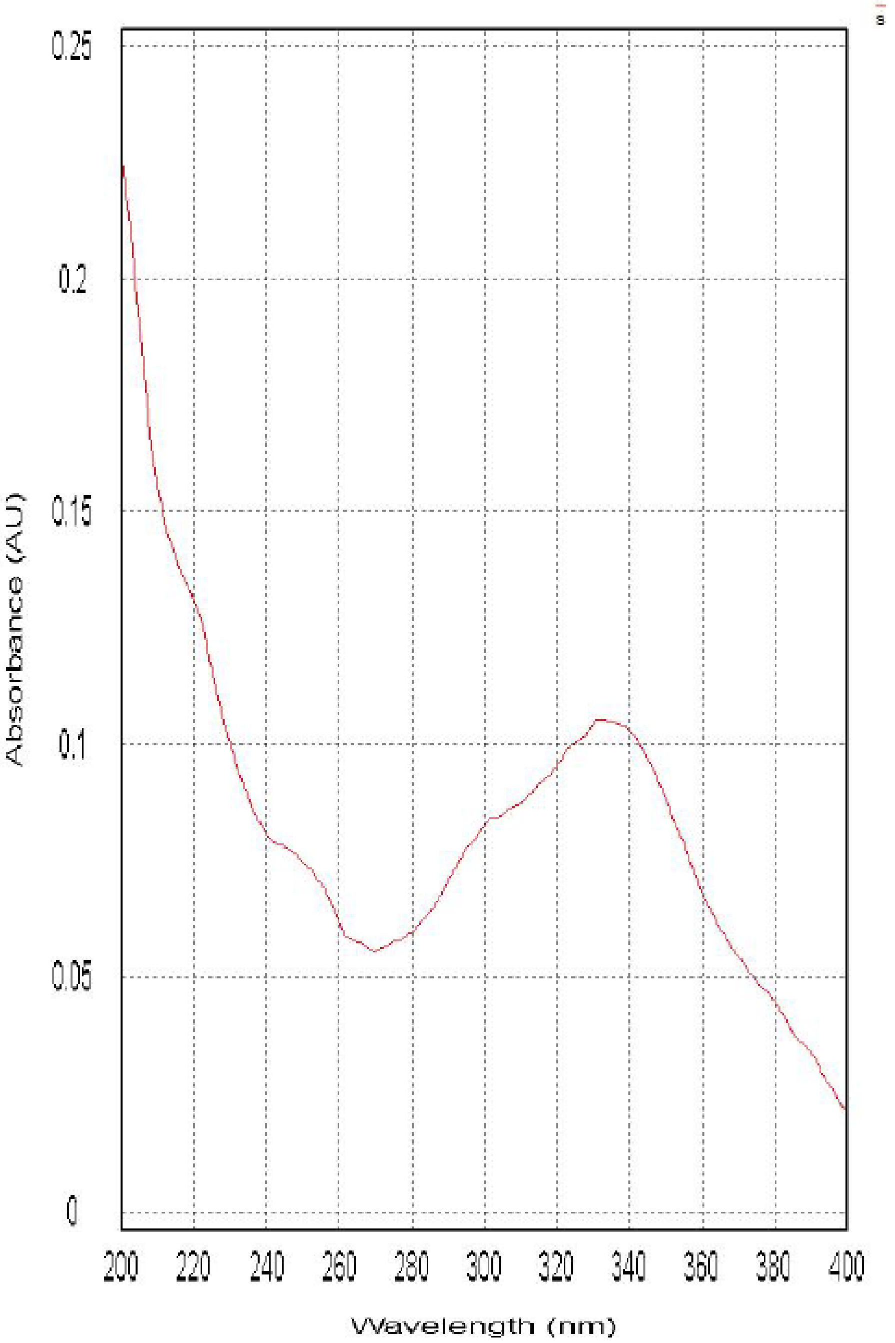

Figure S 3- 1. The HRESIMS of **12**.

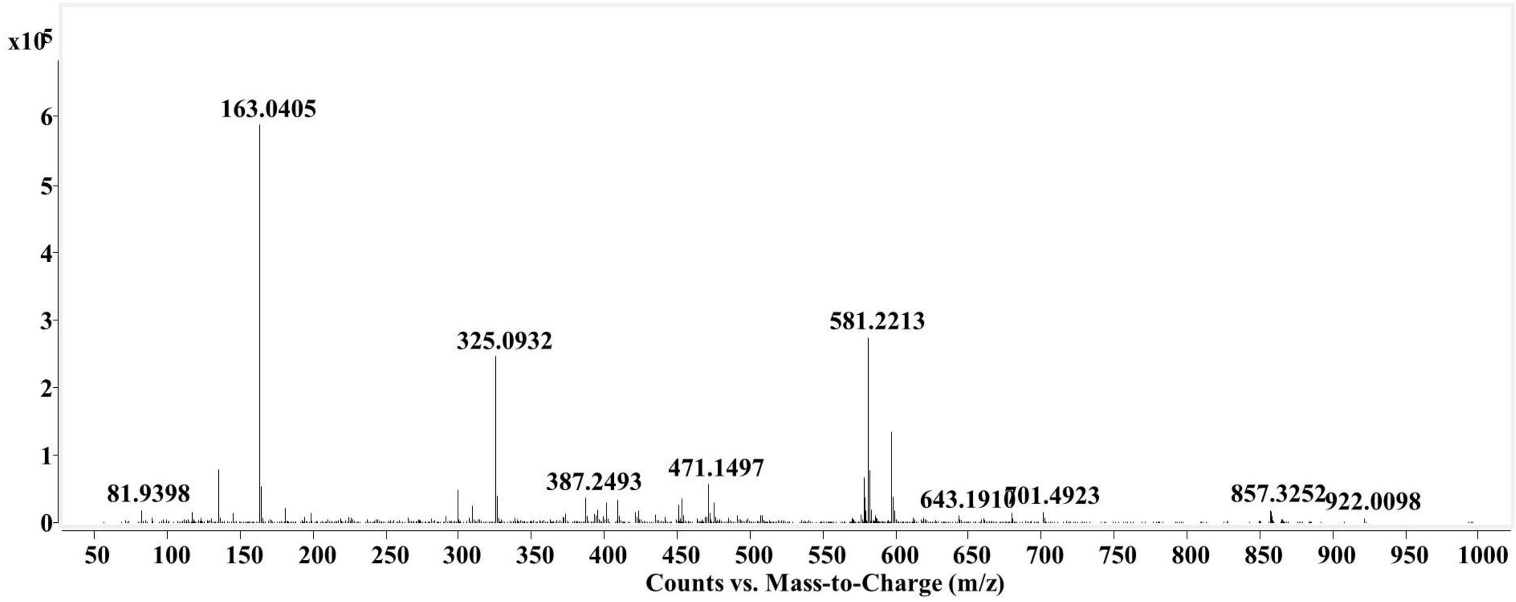

	Formula (M)	Ion Formula	Mass (MFG)	m/z (Calc)	Diff (ppm)
	C24 H39 Na O13	C24 H39 Na2 O13	558.2288	581.2181	-5.81
▶	C26 H38 O13	C26 H38 Na O13	558.2312	581.2205	-1.5
	C42 H31 Na	C42 H31 Na2	558.2323	581.2216	0.48
	C44 H30	C44 H30 Na	558.2348	581.224	4.79

Figure S 3- 2. The ^{1}H NMR (800 MHz) spectrum of **12** in DMSO-d_6

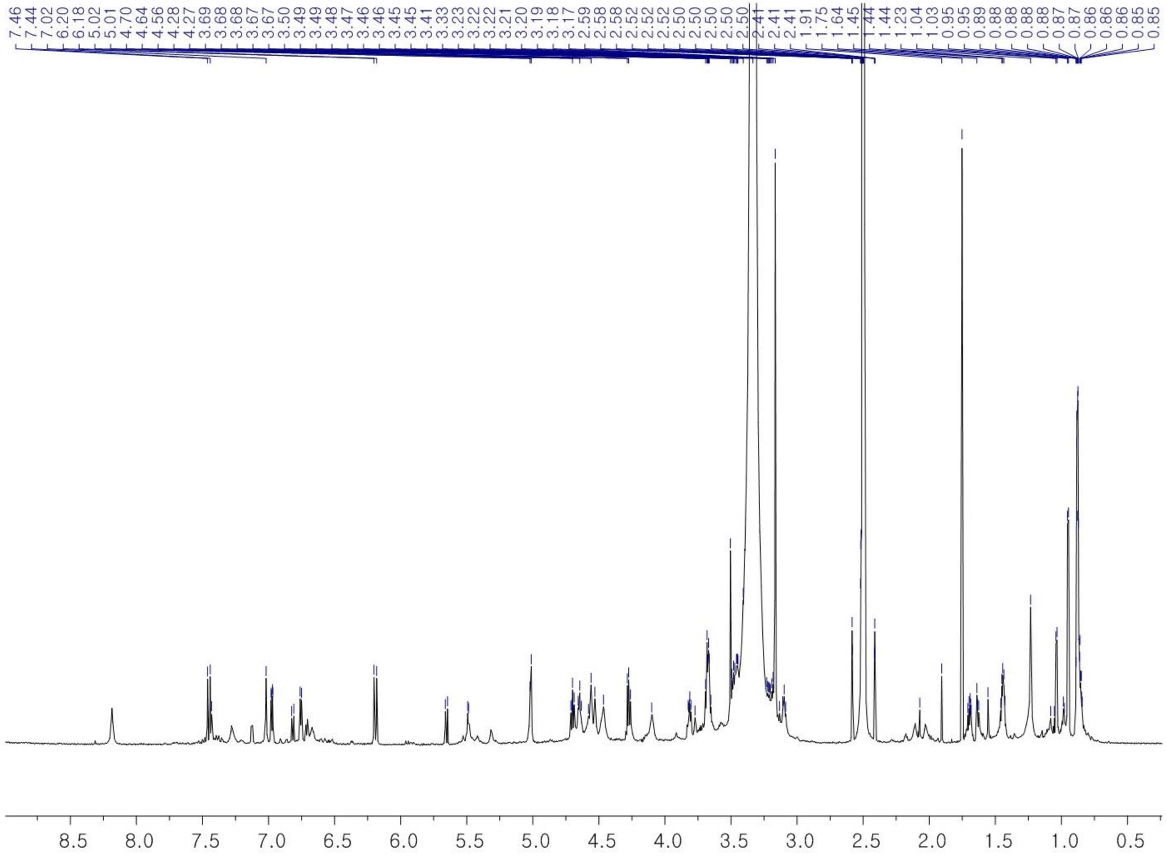

Figure S 3- 3. The ^{13}C NMR (200 MHz) spectrum of **12** in DMSO-d_6

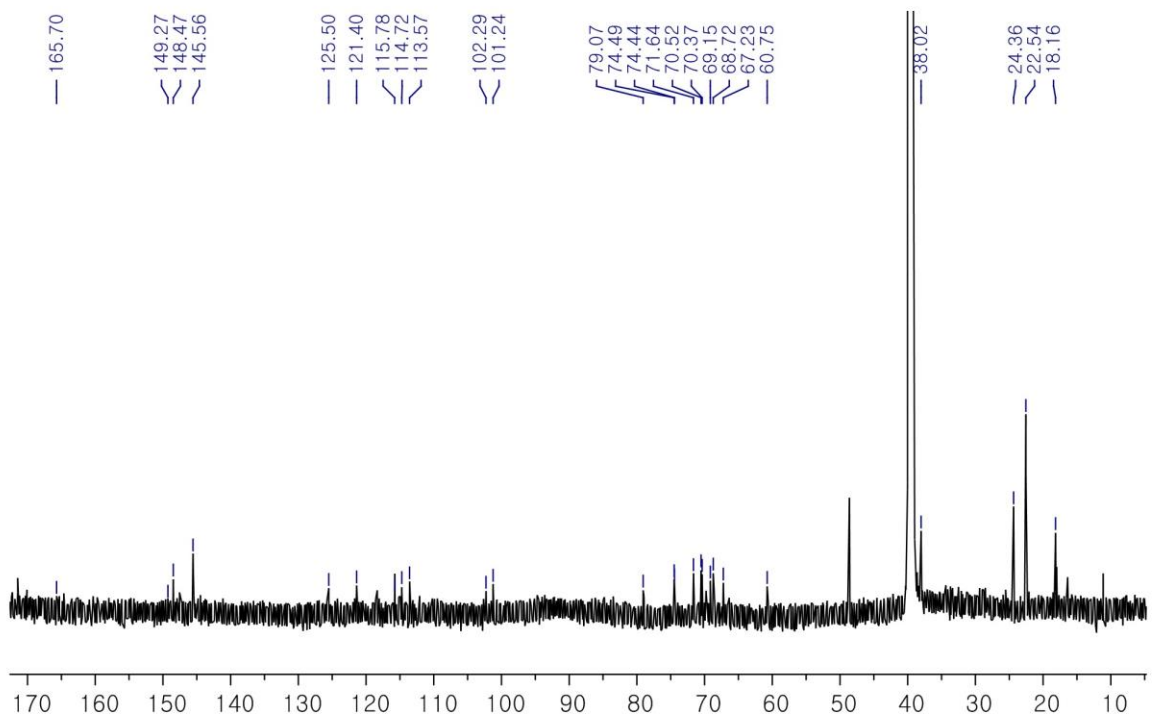

Figure S 3- 4. The HSQC spectrum of **12** in DMSO-d_6

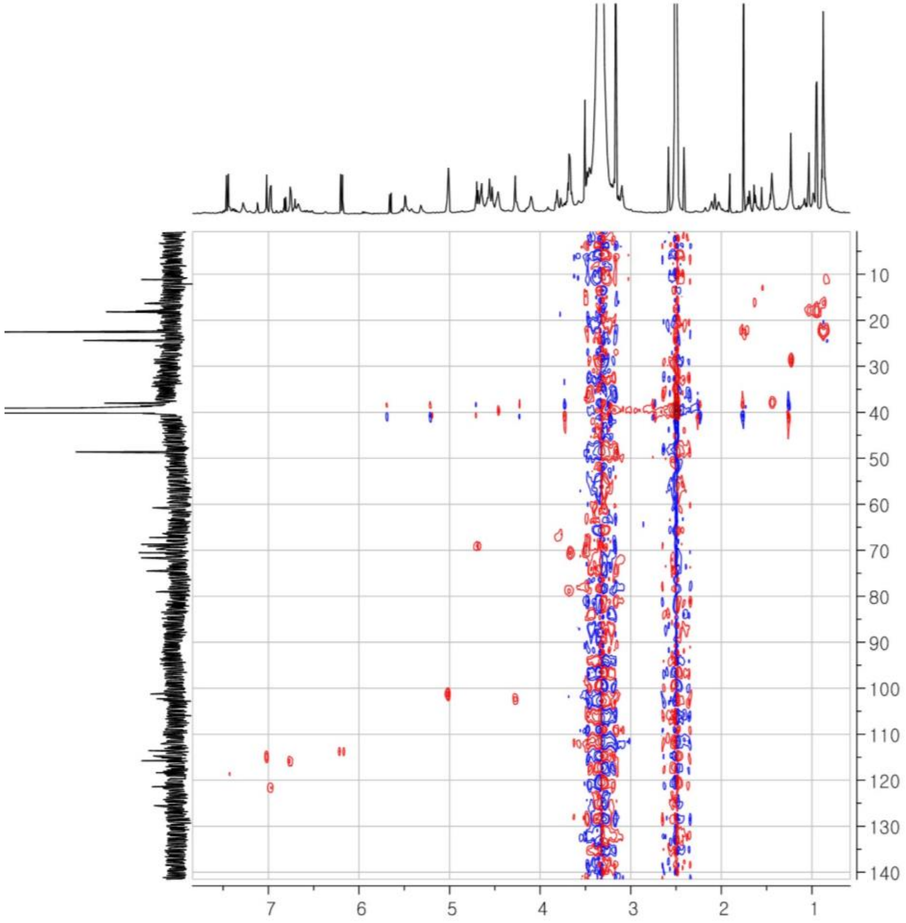

Figure S 3- 5. The COSY spectrum of **12** in DMSO-d_6

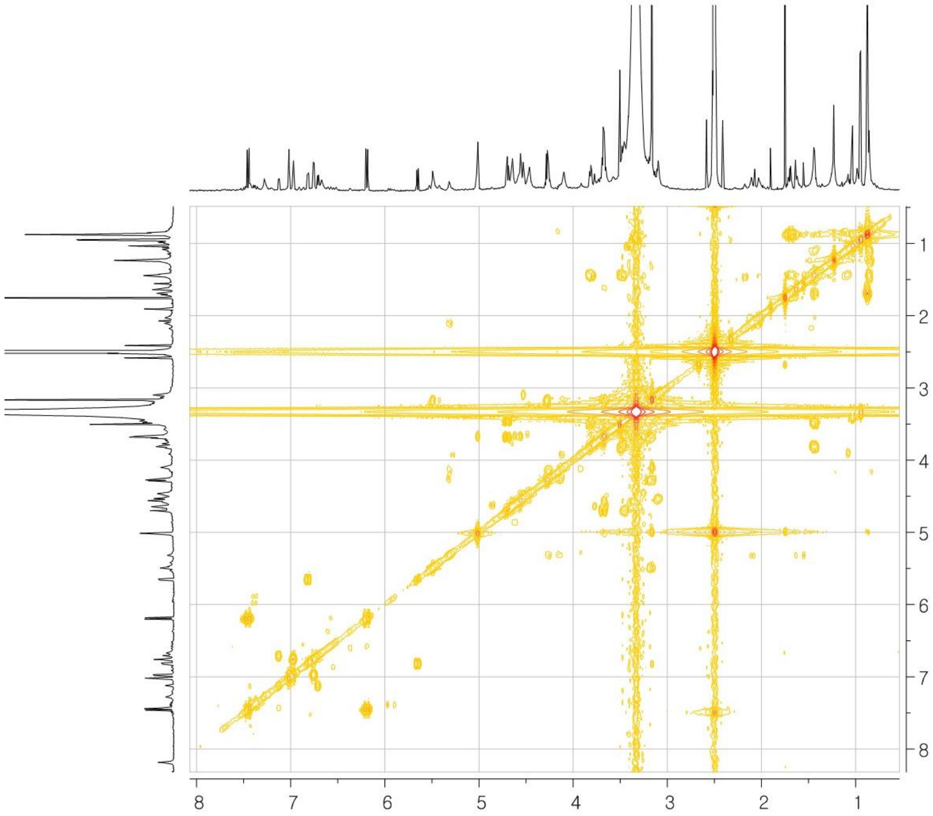

Figure S 3- 6. The HMBC spectrum of **12** in DMSO-d_6

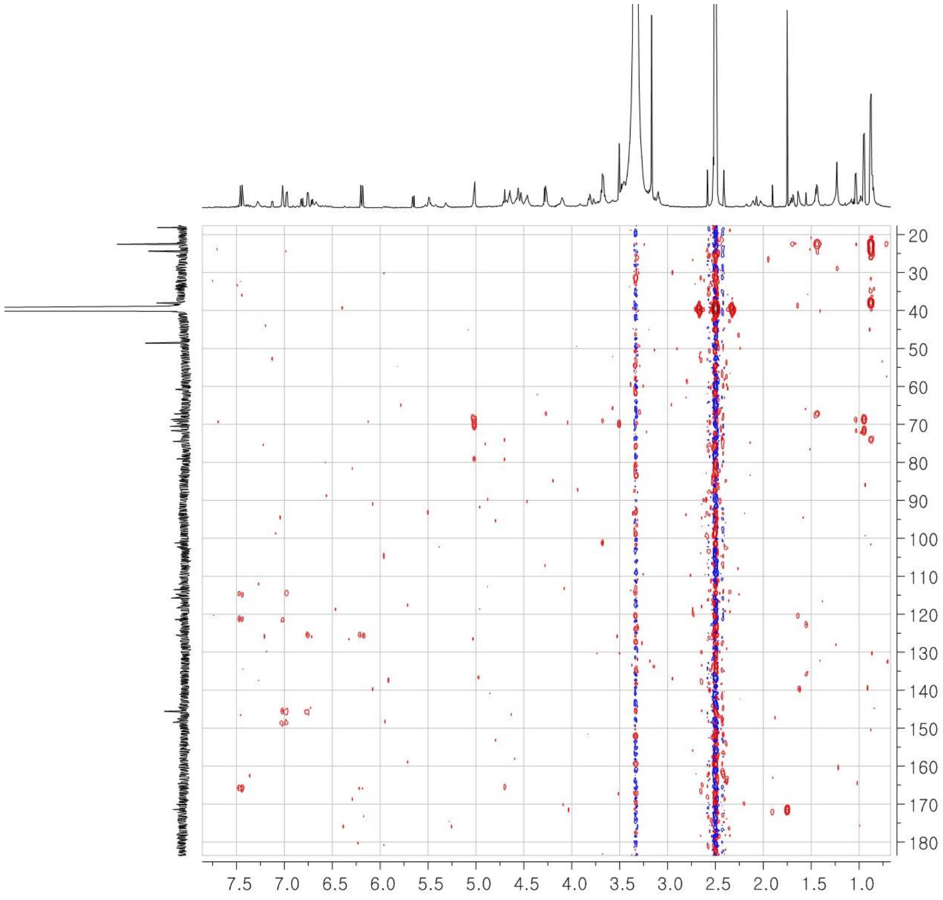

Figure S 3- 7. The UV spectrum of **12**

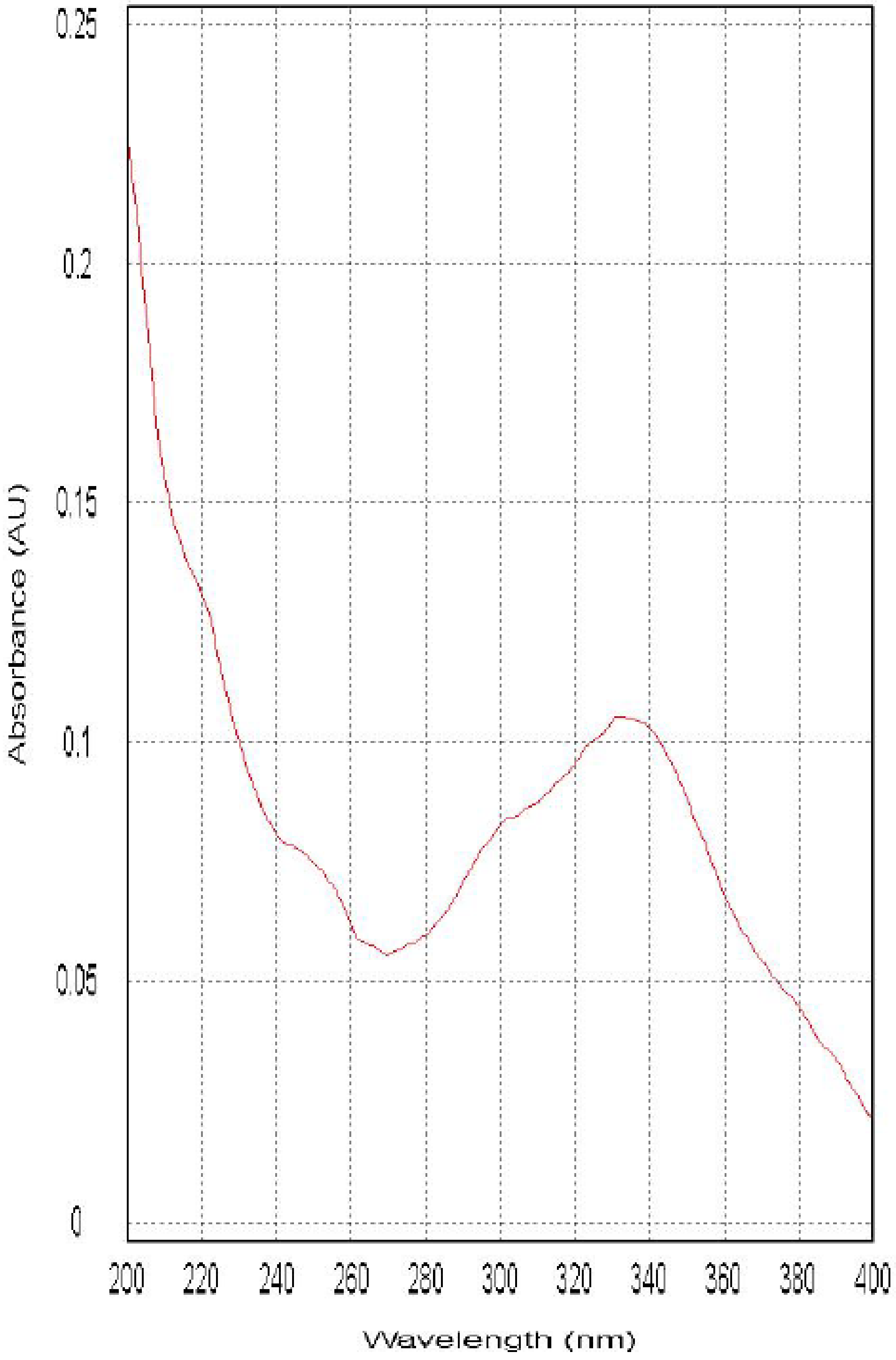

Figure S 4- 1. The HRESIMS of **17**.

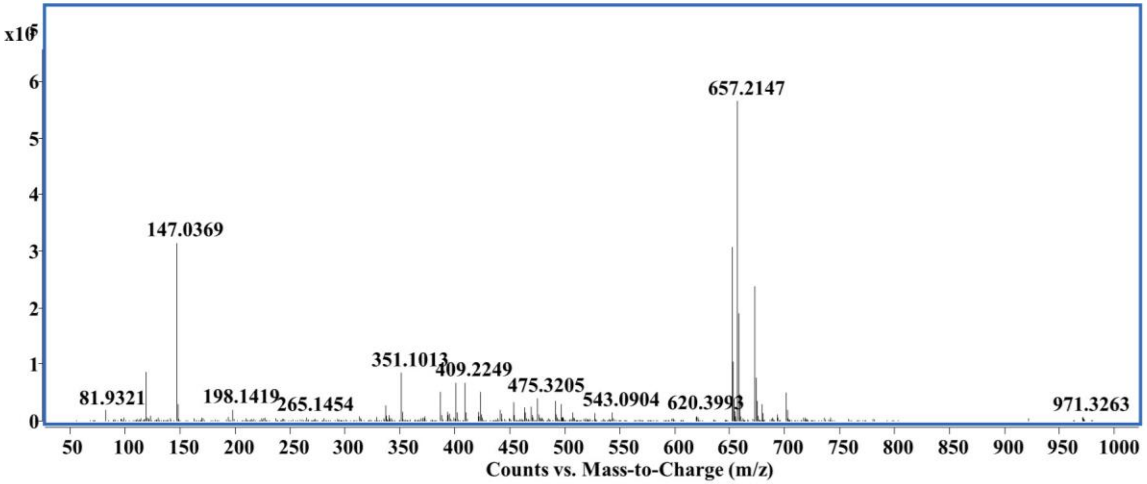

	Formula (M)	Ion Formula	Mass (MFG)	m/z (Calc)	Diff (ppm)
	C29 H39 Na O14	C29 H39 Na2 O14	634.2238	657.213	-2.73
▸	C31 H38 O14	C31 H38 Na O14	634.2262	657.2154	1.07
	C47 H31 Na O	C47 H31 Na2 O	634.2273	657.2165	2.81
	C22 H43 Na O19	C22 H43 Na2 O19	634.2296	657.2188	6.54
	C49 H30 O	C49 H30 Na O	634.2297	657.2189	6.6

Figure S 4- 2. The ^{1}H NMR (300 MHz) spectrum of **17** in DMSO-d_6

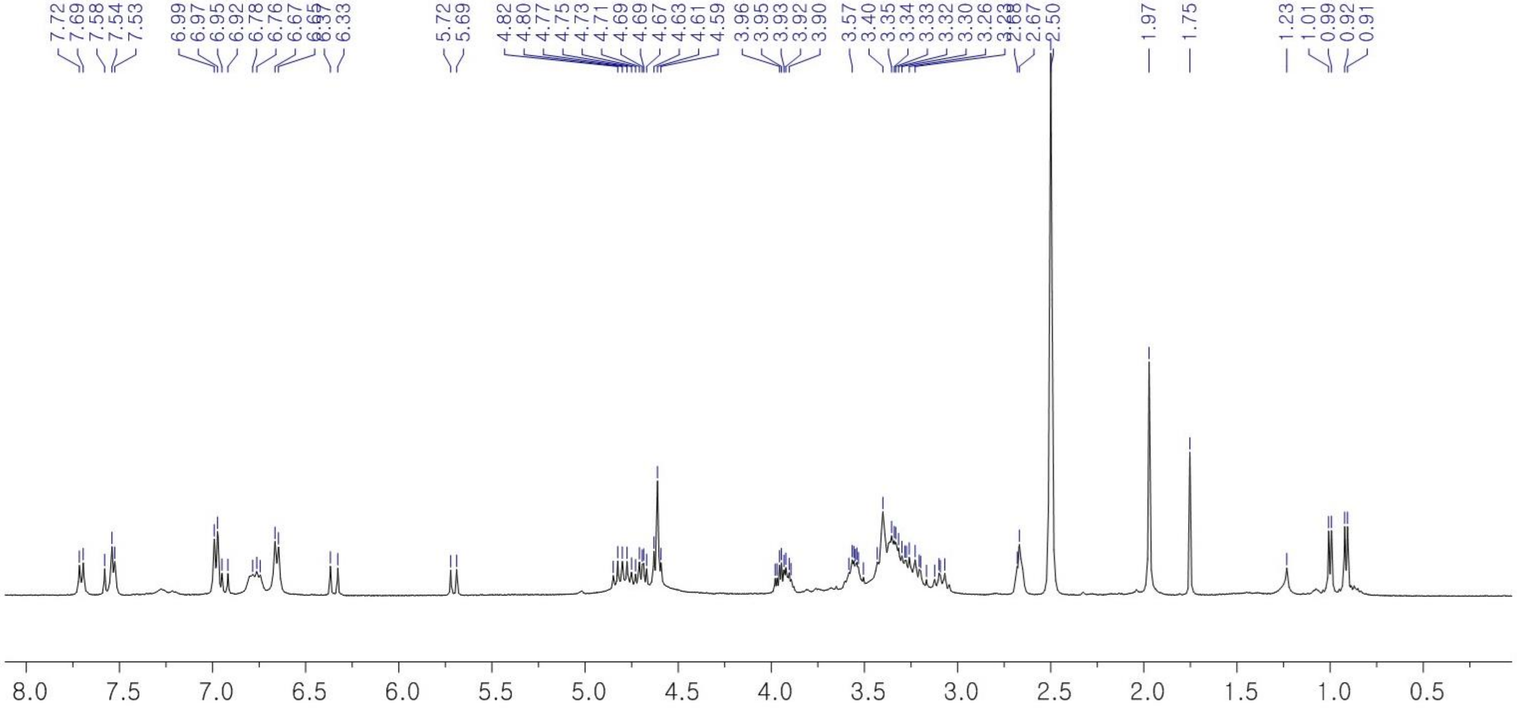

Figure S 4- 3. The ^{13}C NMR (75 MHz) spectrum of **17** in DMSO-d_6

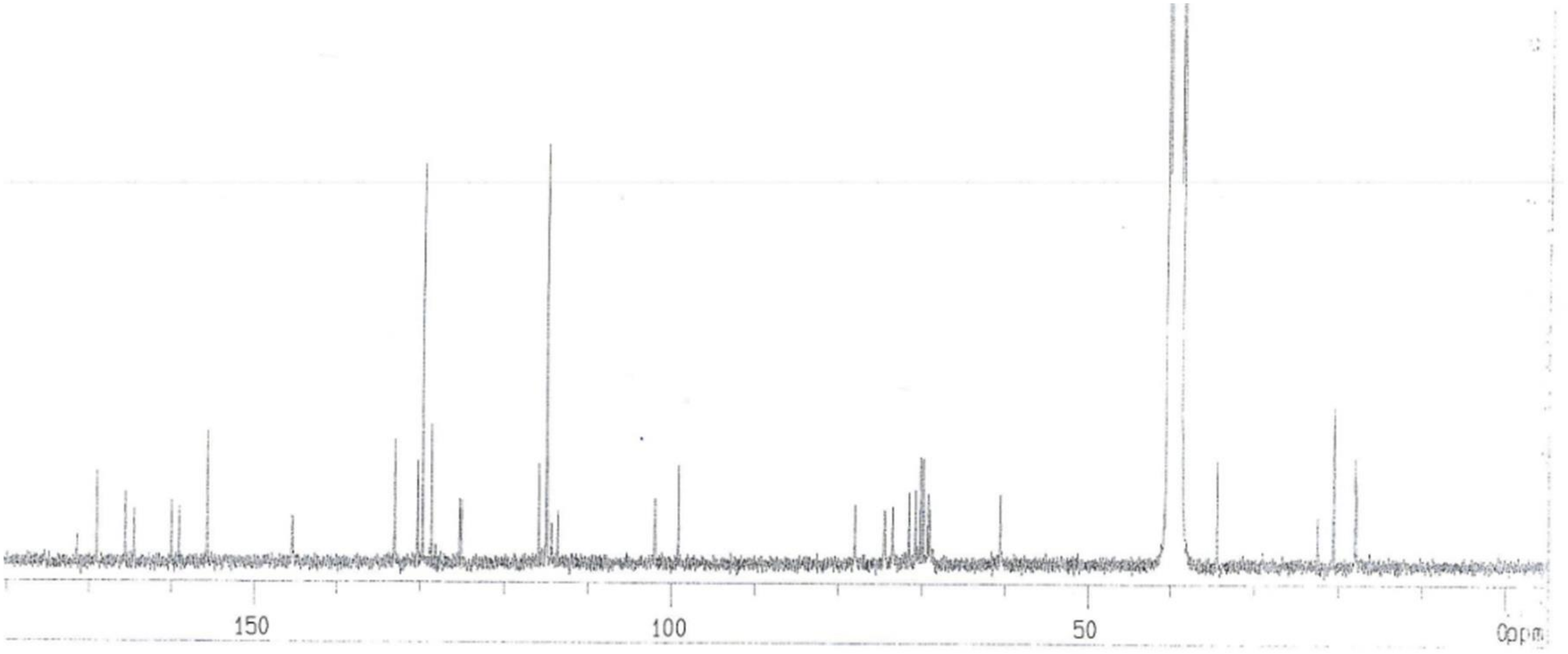

Figure S 4- 4. The HSQC spectrum of **17** in DMSO-d_6

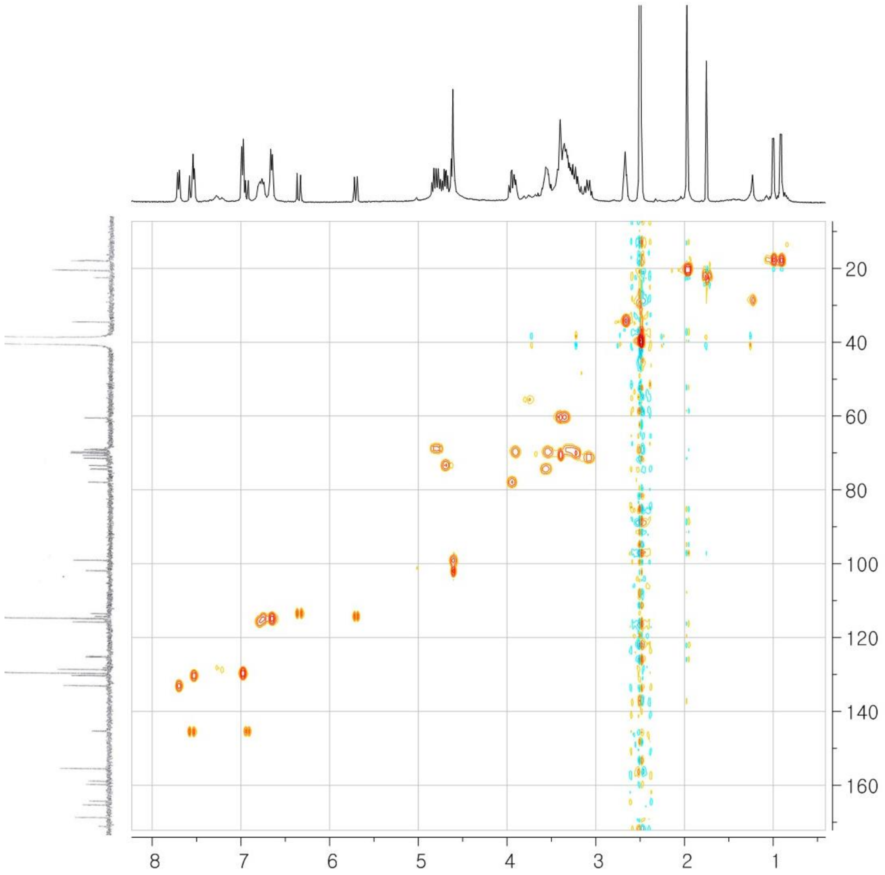

Figure S 4- 5. The COSY spectrum of **17** in DMSO-d_6

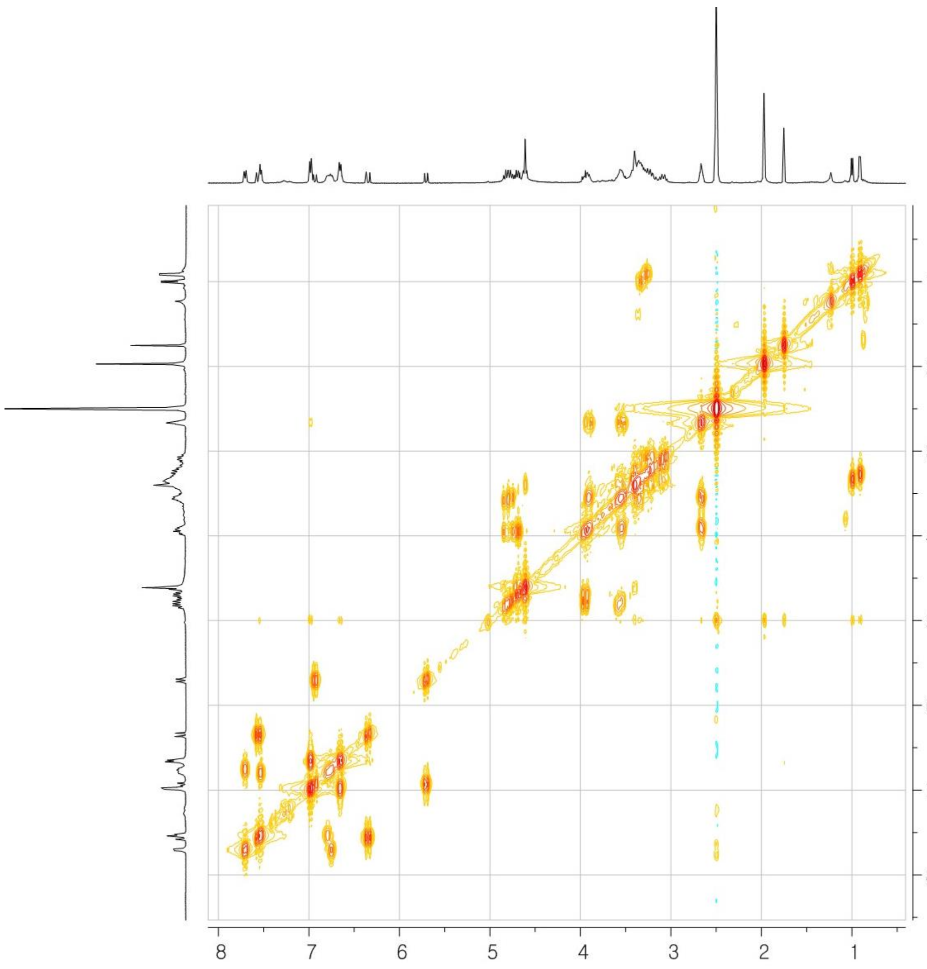

Figure S 4- 6. The HMBC spectrum of **17** in DMSO-d_6

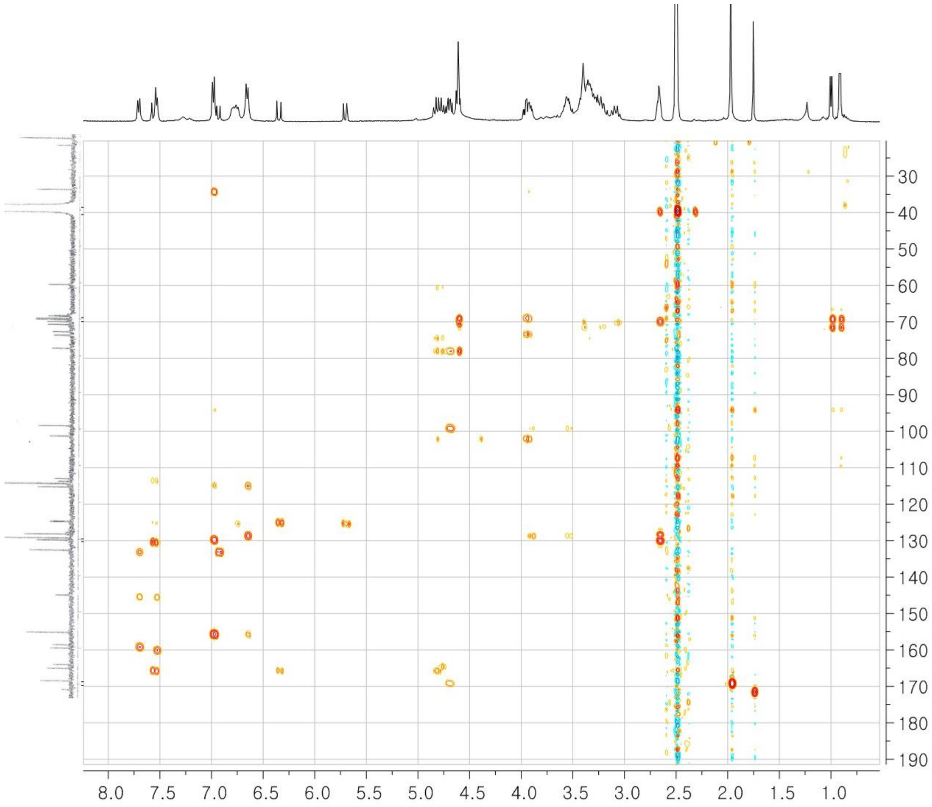

Figure S 4- 7. The UV spectrum of **17**

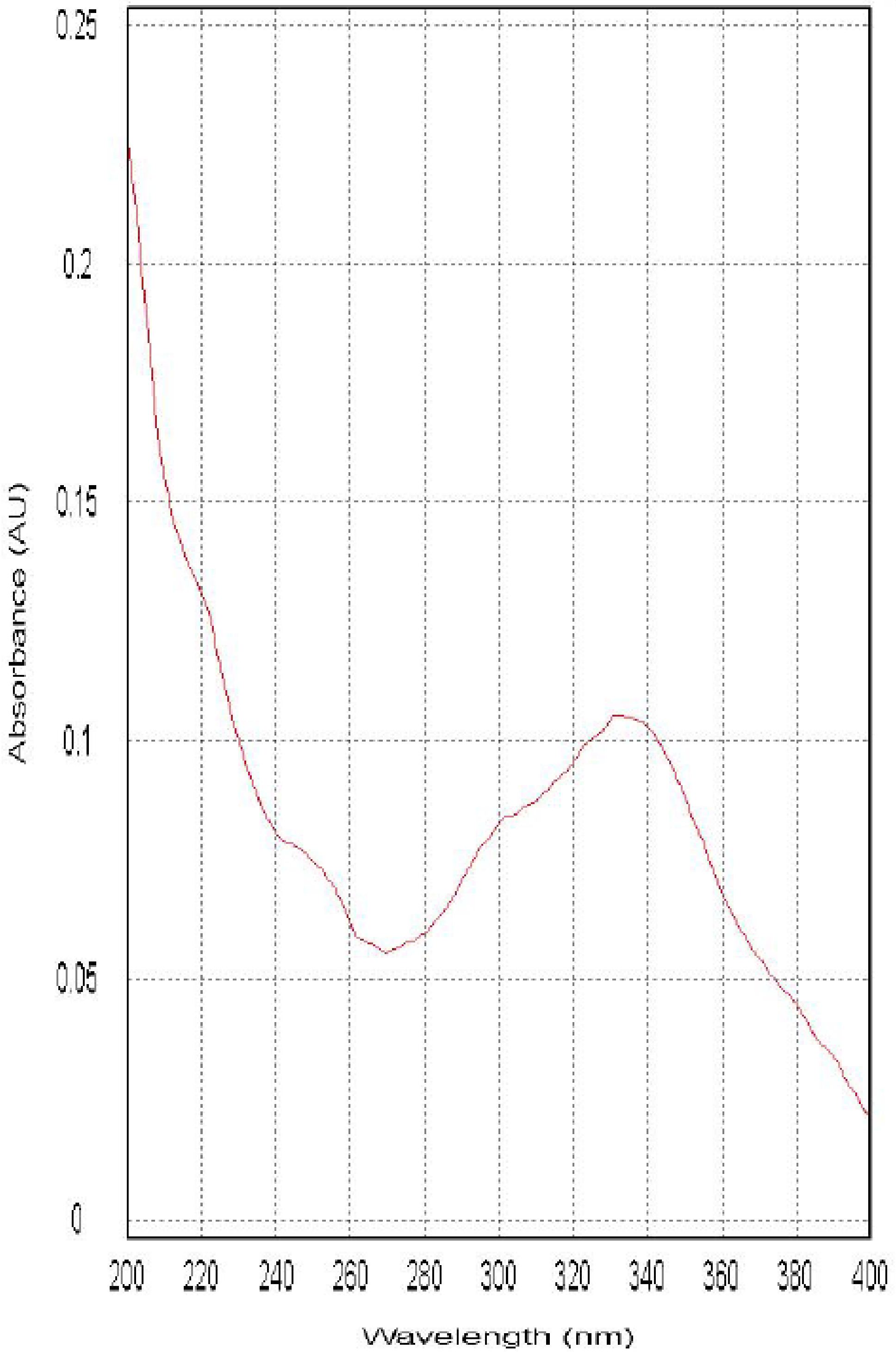

Figure S 5- 1.   The HRESIMS of **18**.

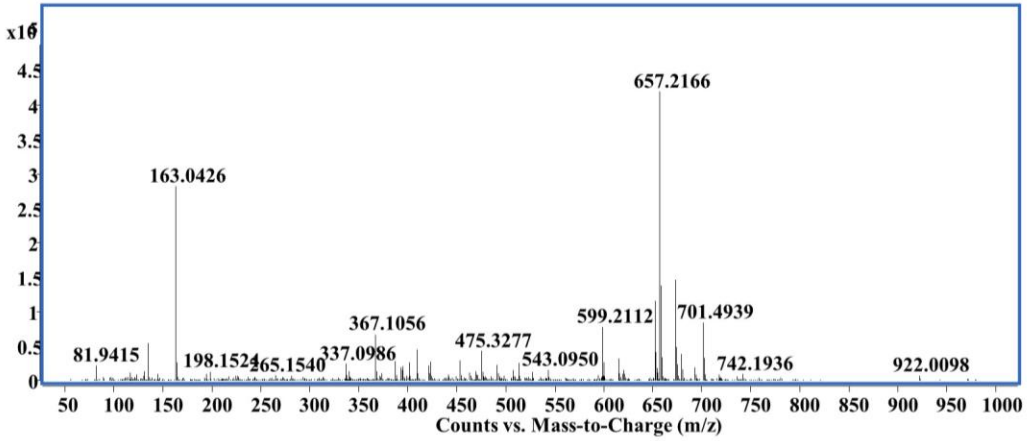

Formula (M)	Ion Formula	Mass (MFG)	m/z (Calc)	Diff (ppm)
C29 H39 Na O14	C29 H39 Na2 O14	634.2238	657.213	-5.72
C31 H38 O14	C31 H38 Na O14	634.2262	657.2154	-1.93
C47 H31 Na O	C47 H31 Na2 O	634.2273	657.2165	-0.19
C22 H43 Na O19	C22 H43 Na2 O19	634.2296	657.2188	3.54
C49 H30 O	C49 H30 Na O	634.2297	657.2189	3.61
C24 H42 O19	C24 H42 Na O19	634.232	657.2213	7.33

Figure S 5- 2. The ^{1}H NMR (800 MHz) spectrum of **18** in DMSO-d_6

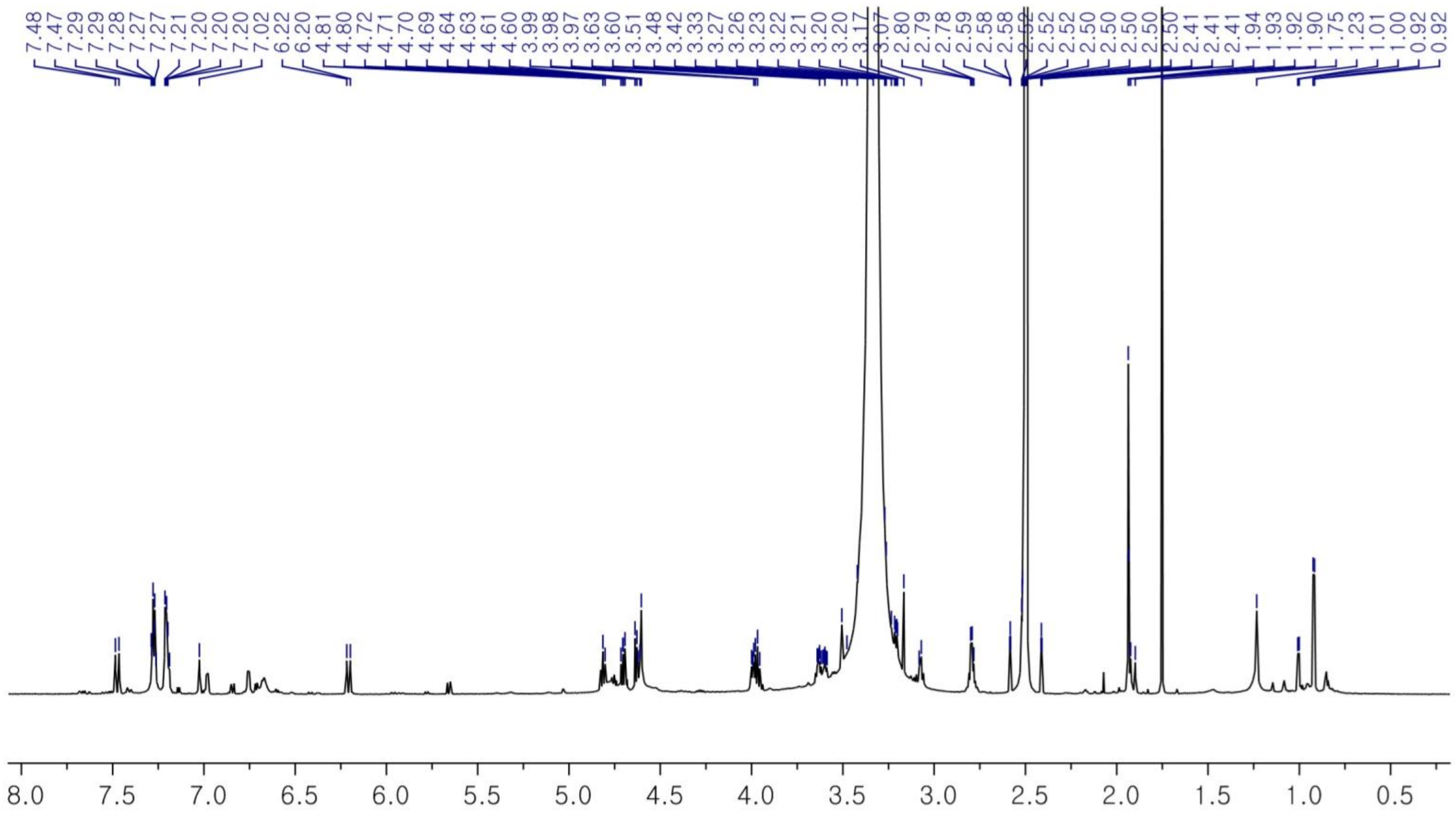

Figure S 5- 3. The ^{13}C NMR (200 MHz) spectrum of **18** in DMSO-d_6

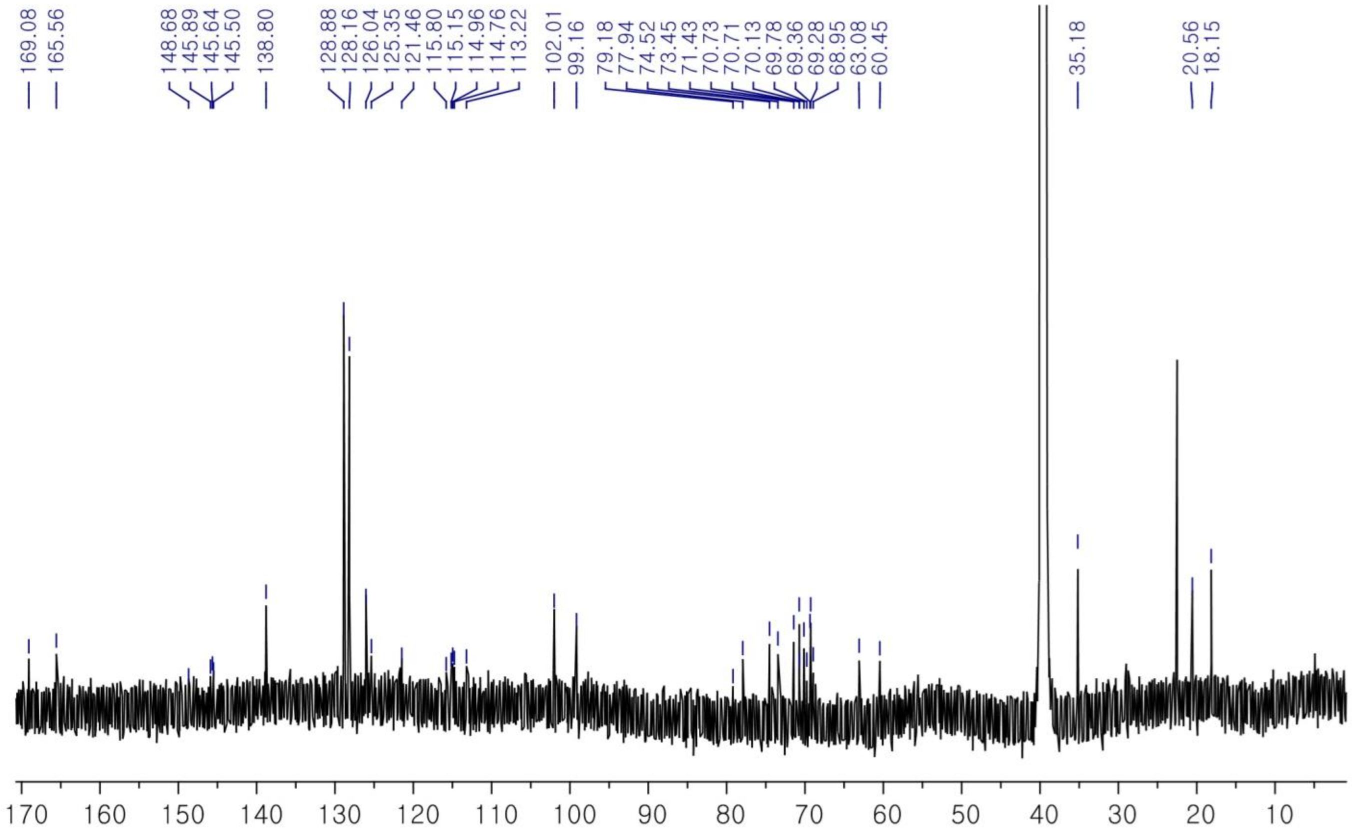

Figure S 5- 4. The HSQC NMR spectrum of **18** in DMSO-d_6

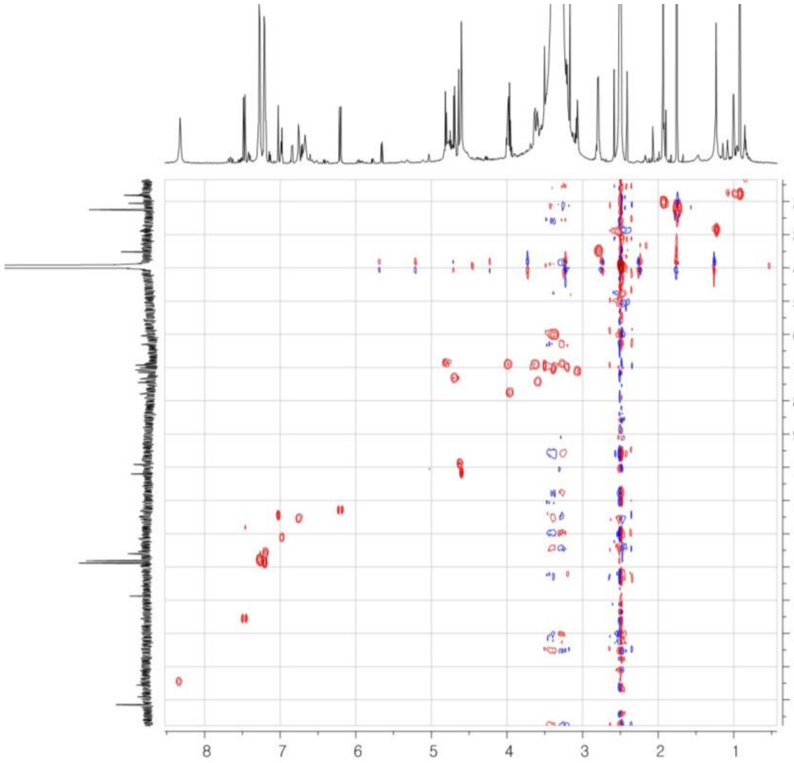

Figure S 5- 5. The COSY NMR spectrum of **18** in DMSO-d_6

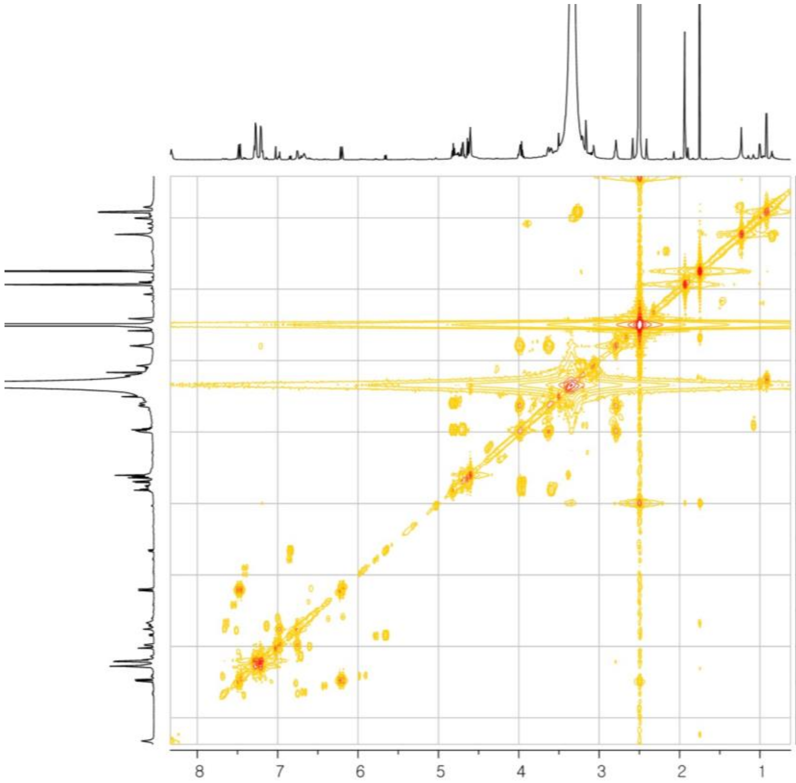

Figure S 5- 6. The HMBC NMR spectrum of **18** in DMSO-d_6

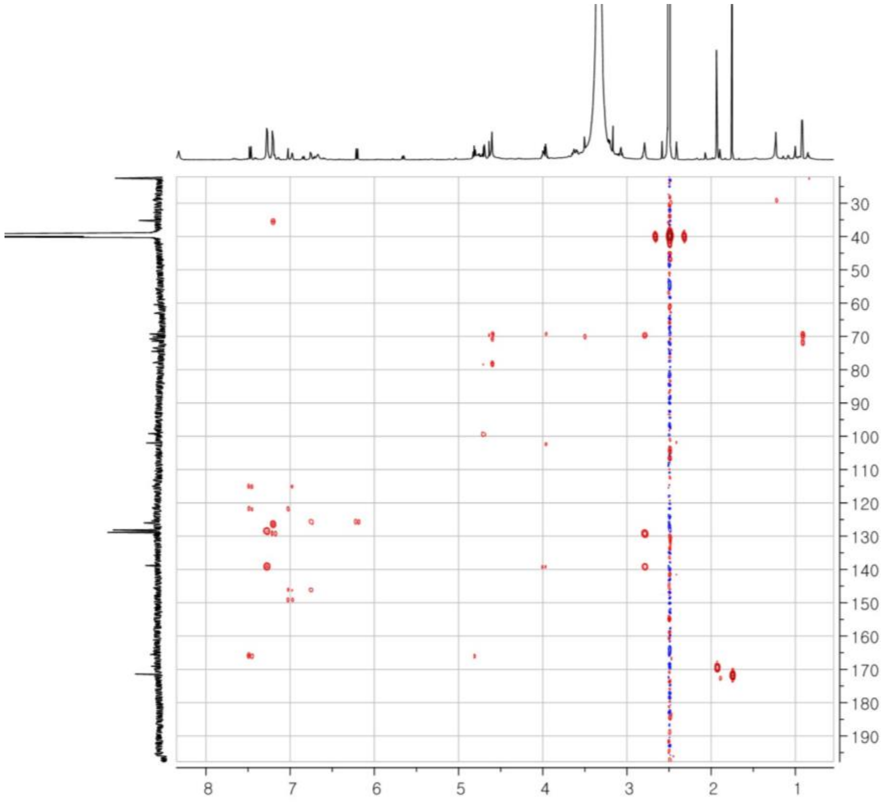

Figure S 5- 7. The UV spectrum of **18**

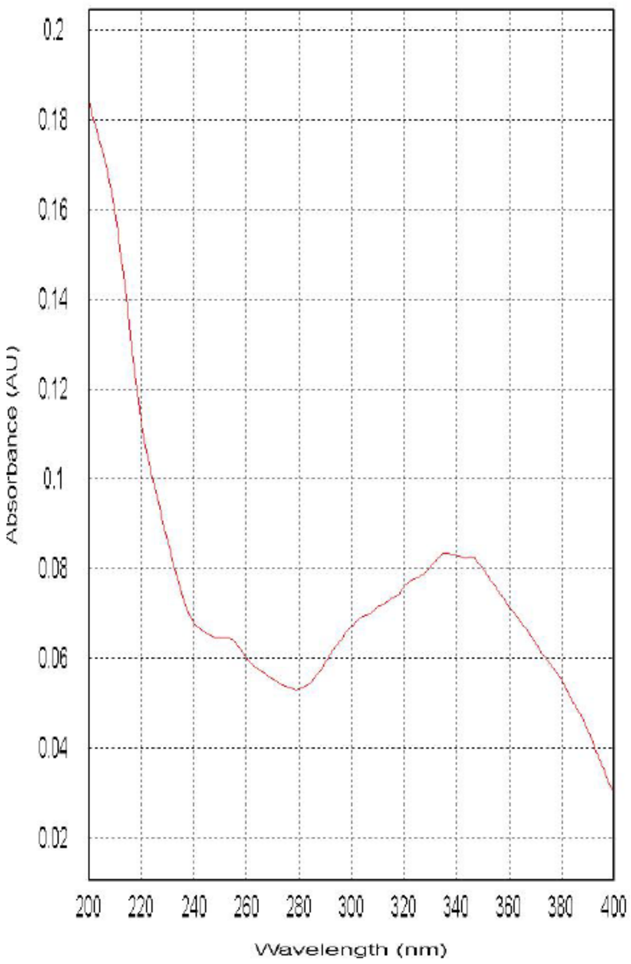

Figure S6- 1. Effects of compounds on the NO production and cell viability of RAW 264.7 cells.

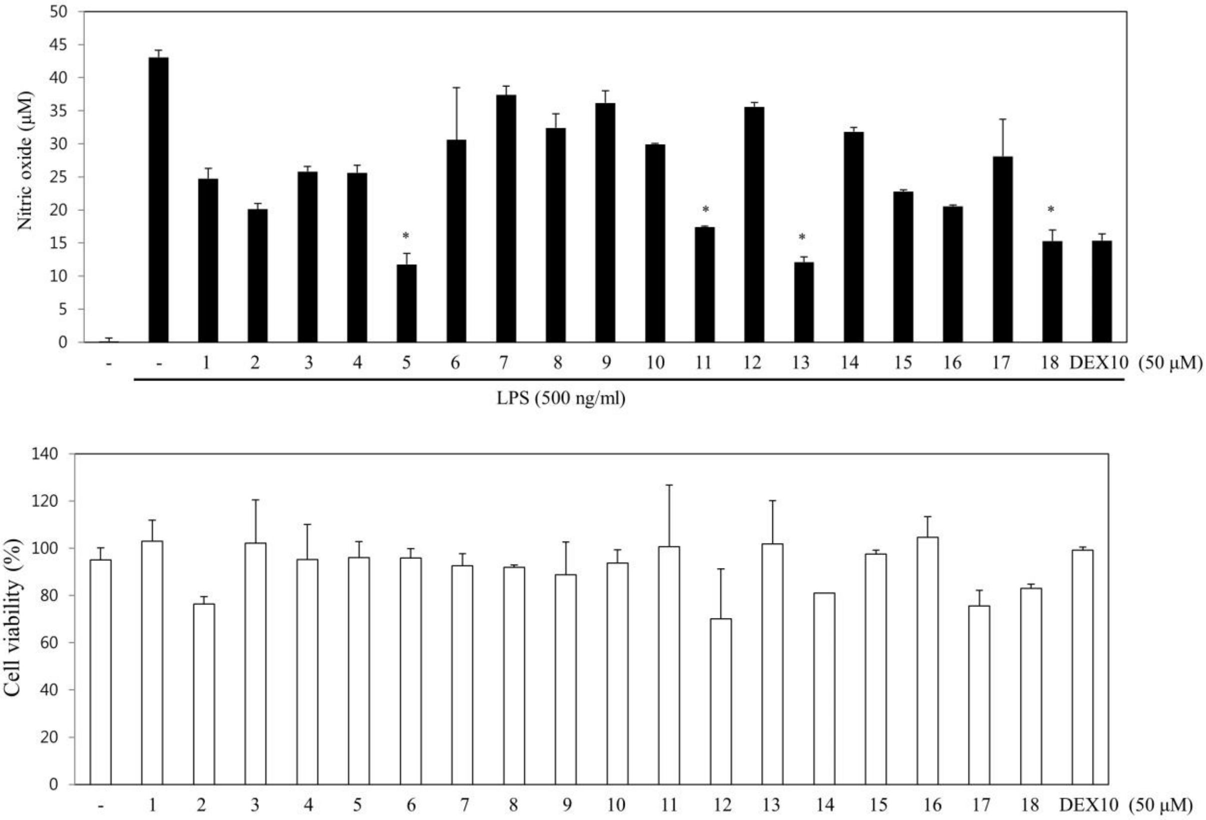

(A) Cells cultured in phenol red- and serum-free media were pretreated with each compound for 30 min and then stimulated with 500 ng/ml final concentration LPS for 24 h. In the culture medium, NO production was measured based on the Griess reaction, as described in the Materials and Methods section. * significant difference from LPS cells, $p < 0.05$. (B) Cells grown in serum-free media were treated with 50 μM of each compound for 24 h, and cell viability was assessed by MTT assay, as described in the Materials and Methods section. Results of independent experiments were averaged and are shown as the percentage of cell viability compared with the viability of normal control cells. Results of independent experiments were averaged and are shown as the percentage of cell viability compared with the viability of solvent control cells.

Figure S6- 2. Dose-response curves of compounds **5**, **11**, **13** and **18**

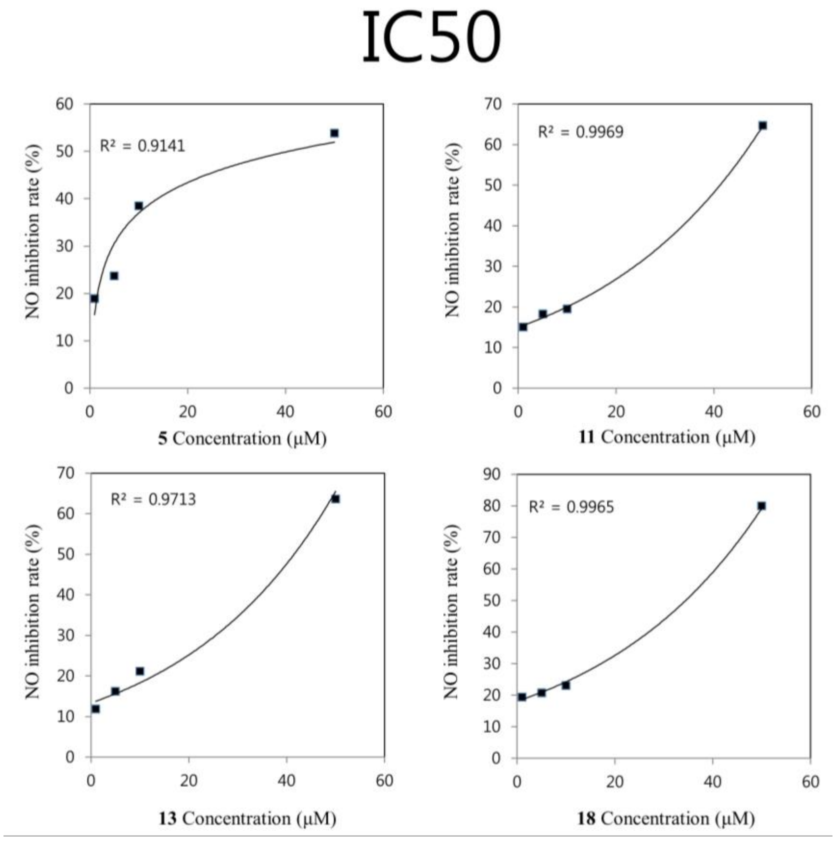

Supplement: Supplementary file 1 [file molecules-22-01138-s001.pdf]
